# Allosteric Inhibitors of SARS‐CoV‐2 RNA‐Dependent RNA Polymerase Based on *N*,*N′*‐Diphenylurea

**DOI:** 10.1002/cmdc.202500644

**Published:** 2025-11-22

**Authors:** Artem Chayka, Matěj Danda, Alžběta Dostálková, Vojtěch Spiwok, Lamija Zijadic, Anna Klimešová, Marina Kapisheva, Michala Zgarbová, Jan Weber, Tomáš Ruml, Michaela Rumlová, Zlatko Janeba

**Affiliations:** ^1^ Institute of Organic Chemistry and Biochemistry of the Czech Academy of Sciences Flemingovo nám. 2 Prague 6 160 00 Czech Republic; ^2^ Department of Biotechnology University of Chemistry and Technology, Prague Technická 5 Prague 6 166 28 Czech Republic; ^3^ Department of Biochemistry and Microbiology University of Chemistry and Technology, Prague Technická 5 Prague 6 166 28 Czech Republic; ^4^ Department of Genetics and Microbiology Faculty of Sciences Charles University Viničná 5 Prague 2 128 44 Czech Republic

**Keywords:** allosteric inhibitor, kinetic solubility, RdRp, SARS‐CoV‐2

## Abstract

The COVID‐19 pandemic, caused by the highly transmissible SARS‐CoV‐2 virus, has highlighted the urgent need for effective small‐molecule antivirals. To date, only a few such agents, including molnupiravir and remdesivir, have been approved by the FDA. In our previous study, a novel class of SARS‐CoV‐2 RNA‐dependent RNA polymerase (RdRp) inhibitors based on an *N*,*N*′‐diphenylurea scaffold was identified; however, these compounds exhibited poor aqueous solubility and significant cytotoxicity. Herein, the design, synthesis, and evaluation of twenty‐seven new derivatives aimed at improving solubility and reducing cytotoxicity through targeted scaffold modifications are reported. Seven analogs display enhanced aqueous solubility (kinetic solubility > 10 µM), and nine compounds show residual RdRp activity (RA—determined at 10 μM concentration of screened compounds) below 50%, with the most potent analog achieving an RA value of 34%. Despite these improvements, cytotoxicity remains a limitation across the series. These findings provide valuable structure–activity relationship insights and direct ongoing optimization efforts toward developing less toxic, soluble RdRp inhibitors with improved antiviral profiles.

## Graphical abstract

Inhibitors of SARS‐CoV‐2 RdRp activity based on *N*,*N′*‐diphenylurea scaffold have been identified. Several compounds exhibit residual activity (RA) below 50% in an enzymatic assay (at 10 µM concentration) and improved aqueous solubility compared to the previously reported series of inhibitors.

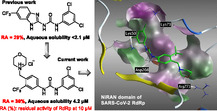

## 1 Introduction

In late 2019, SARS‐CoV‐2 sparked the COVID‐19 pandemic, which remains a significant health challenge worldwide, despite approved treatments and reduced severity of newer variants.^[^ ^1^, ^2^, ^3^ ^]^ One key approach in antiviral drug development targets essential viral enzymes,^[^ ^4^ ^]^ such as the SARS‐CoV‐2 RNA‐dependent RNA polymerase (RdRp), which is crucial for viral replication and is absent in humans.^[^ ^5^ ^]^ RdRp inhibitors fall into two categories: catalytic site inhibitors, which include broad‐spectrum drugs like favipiravir, remdesivir, and molnupiravir,^[^ ^6^ ^,^ ^7^ ^]^ and allosteric inhibitors, which have not yet been successfully developed for SARS‐CoV‐2.^[^ ^8^ ^]^

While catalytic site inhibitors show mixed effectiveness because of the proofreading activity of the RdRp complex,^[^ ^9^ ^]^ allosteric inhibitors hold promise to counteract resistance. In this context, we have recently identified potential allosteric sites on RdRp, screened available compounds, and discovered compounds active in enzymatic and cell‐based assays as potential SARS‐CoV‐2 RdRp allosteric inhibitors.^[^ ^10^ ^]^

**Figure 1:**
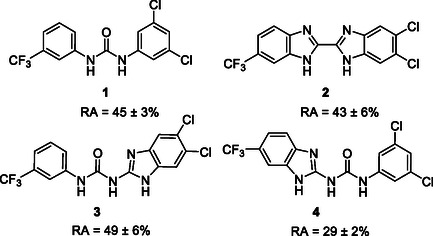
Structures of compounds **1**–**4**.^[^ ^10^ ^]^

One of the most promising compounds was 1‐(3,5‐dichlorophenyl)‐3‐(3‐(trifluoromethyl)phenyl)urea (**1**, **Figure** **1**), which reduced residual activity of the RdRp in the enzymatic assay to 45%.^[^ ^10^ ^]^ The residual activity (RA, in %) of the SARS‐CoV‐2 RdRp complex is determined at 10 μM concentration of screened compound in an RNA polymerase assay established in our previous work^[^ ^10^ ^]^ and is calculated as the area under the amplification curve (AUC) of the reaction in the presence of the compound divided by the AUC of the noninhibited reaction. The core structure of compound **1** was found to be interchangeable with electronically similar structures such as 2,2′‐bisimidazol (used in compound **2** with RA = 43 ± 6%) and (1*H*‐benzimidazol‐2‐yl)urea (in compounds **3** and **4**, with RA = 49 ± 6% and 29 ± 2%, respectively) to preserve the inhibitory activity.^[^ ^10^ ^]^

However, all previously reported compounds exhibited high cytotoxicity in cell‐based assays and poor aqueous solubility (kinetic solubility < 10 µM).^[^ ^10^ ^]^ To overcome these limitations, further structural optimization of compound **1** (Figure 1) was undertaken. This compound was selected as a suitable lead for modification due to its favorable inhibitory activity in the enzymatic assay, relatively low molecular weight (349.1 g mol^−1^), and modest synthetic complexity, making it an attractive starting point for systematic optimization.

## 2 Results and Discussion

In general, compound solubility can be enhanced through several structural strategies.^[^ ^11^ ^]^ 1) Distortion of molecular planarity: highly planar molecules tend to pack efficiently into crystals, thereby reducing solubility. 2) Incorporation of heteroaromatic rings: replacement of benzene with heteroaromatic systems such as pyridine can disrupt crystal packing and improve solvation. 3) Introduction of solubilizing functionalities: polar substituents, such as carboxamide moiety, were evaluated for their solubilizing effects. 4) Incorporation of protonatable groups: selected basic groups were introduced *via* methylene linkers to prevent excessive electron donation into the aromatic system, which could otherwise attenuate biological activity.^[^ ^10^ ^]^

All of these strategies were systematically explored in the design of the new compounds described herein.

### 2.1 Synthesis of the First Series of Compounds

**Scheme 1:**
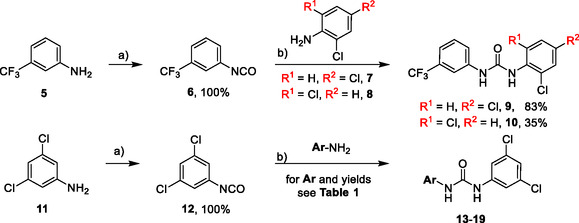
Synthesis of compounds **9**, **10**, **13**–**19**. Reagents and conditions: a) triphosgene, Et_3_N, DCM, 0 °C‐RT; b) DCM, 60 °C.

Compounds with distorted planarity, i.e., derivatives **9**, **10**, **13**, and **14**, as well as compounds containing heteroaromatic ring, i.e., compounds **15–19**, were prepared by the reaction of isocyanates **6** and **12** with corresponding anilines (**Scheme** **1**, **Table** **1**).

**Table 1:** Structures and yields of *N*,*N′*‐diphenylurea derivatives **13**–**19** (Scheme 1).

Compound	Ar	Yield [%]	Compound	Ar	Yield [%]
**13**	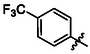	82	**17**	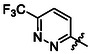	46
**14**	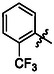	77	**18**	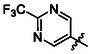	99
**15**	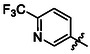	86	**19**	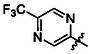	35
**16**	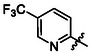	19			

**Scheme 2:**
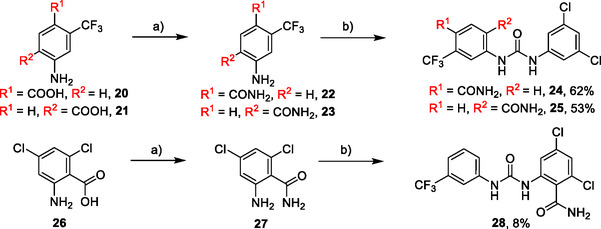
Synthesis of compounds **24**, **25**, and **28**. Reagents and conditions: a) DBU, HATU, NH_4_Cl, DCM, RT; b) corresponding isocyanate, DCM, 60 °C.

Synthesis of compounds bearing carboxamide moiety, derivatives **24**, **25**, and **28** (**Scheme** **2**), started from commercially available aminobenzoic acids **20**, **21**, and **26**, which were treated with NH_4_Cl/HATU/DBU and subsequently with the corresponding isocyanate.

**Scheme 3:**
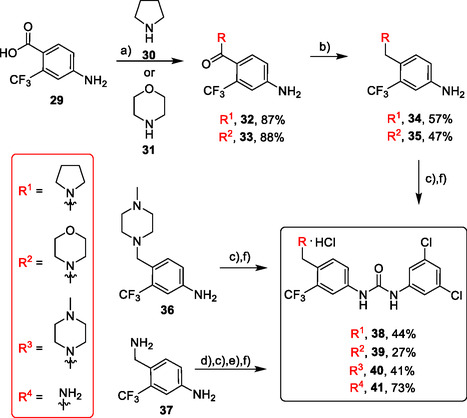
Synthesis of compound **38–41**. Reagents and conditions: a) Et_3_N, HATU, DCM, RT; b) LiAlH_4_ THF, 50 °C; c) **12**, DCM, 60 °C; d) Boc_2_O, Et_3_N, THF, RT; e) TFA, RT; f) HCl, dioxane, RT.

Compounds **38** and **39** (**Scheme** **3**) were prepared from **29**, which was coupled with pyrrolidine (**30**) or morpholine (**31**) to give corresponding amides **32** and **33** in high yields. The amides were reduced with LiAlH_4_ to give tertiary amines **34** and **35**, which reacted in the next step with isocyanate **12** to give the final compounds **38** and **39**. Synthesis of compound **40** was carried out by the reaction of commercially available aniline **36** and isocyanate **12**. Compound **41** was synthesized from commercially available benzylamine **37**, which was protected using the Boc group and treated with isocyanate **12** to give the final compound after the Boc group removal with TFA. Compounds **38–41** were isolated as HCl salts to improve their aqueous solubility.

**Scheme 4:**
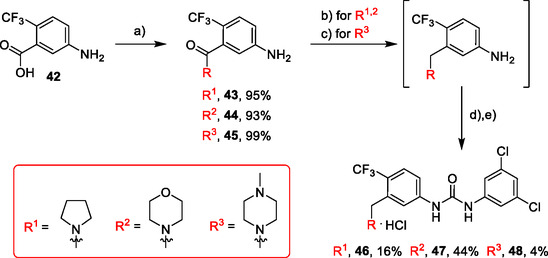
Synthesis of compounds **46–48**. Reagents and conditions: a) Et_3_N, HATU, DCM, RT; b) synhydride, THF, 50 °C; c) LiAlH_4_, THF, 50 °C; d) **12**, DCM, 60 °C; e) HCl, dioxane, RT.

Compounds **46–48** (**Scheme** **4**) were prepared in a similar way as described above. Amides **43–45** (prepared from **42**) were reduced into the corresponding tertiary amines with synhydride (note: using LiAlH_4_ afforded poor yields). The amines were treated with isocyanate **12** to give final products (isolated as HCl salts).

**Scheme 5:**
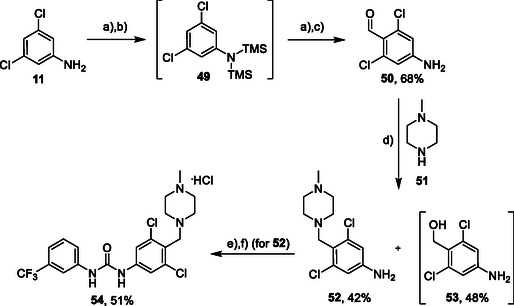
Synthesis of compound **54**. Reagents and conditions: a) BuLi, THF, −78 °C; b) TMSCl, −20 °C; c) DMF, −20 °C; d) NaBH_3_CN, MeOH, RT; e) **6**, DCM, 60 °C; f) HCl, dioxane, RT.

The introduction of (4‐methylpiperazyl)methyl moiety to 3,5‐dichloroaniline proved complicated. After several attempts, metalation of the substrate with dynamic protection of the amino group^[^ ^12^ ^]^ proved to be successful. Dynamic protection of the amino group with two TMS groups was conducted, afterwards *para* position to the amino group was deprotonated with butyllithium and the reaction mixture was quenched with dry DMF, affording the desired intermediate **50**. In the next step, compound **50** reacted with *N*‐methylpiperazine (**51**) under reductive amination conditions to afford compound **52** in a 42% yield. As a side product, alcohol **53** (48%) was isolated. In the last step, compound **52** reacted with isocyanate **6** to give the desired product **54** (as HCl salt) in a 51% yield (**Scheme** **5**).

### 2.2 Biological Activity of the First Series of Compounds

The first series of compounds (derivatives of **1**, Figure 1) can be divided into two groups: derivatives bearing preserved 3,5‐dichlorophenyl ring (**Table** **2**) and derivatives with preserved 3‐(trifluoromethyl)phenyl ring (**Table** **3**). Their resactivity (RA) in enzymatic assay was measured and for selected compounds, additional data were collected (cell‐based assay activity, toxicity, and kinetic solubility).

**Table 2:** Residual activity (RA) of compounds with preserved 3,5‐dichlorophenyl ring, EC_50_ and CC_50_ values of selected compounds in Calu‐3 cells infected with SARS‐CoV‐2, and their kinetic solubility.

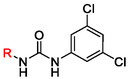					
Compound	R	RA ± SD [%]a)	EC_50_ [µM]b)	CC_50 _± SE [µM]c)	Kinetic solubility [µM]
**13**	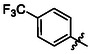	34 ± 14	>0.29	0.29 ± 0.03	<2.1
**14**	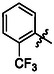	100	–	–	–
**15**	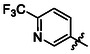	35 ± 9	>1.3	1.3 ± 0.13	3.7 ± 0,9
**16**	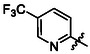	100	–	–	–
**17**	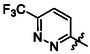	68 ± 9	–	–	–
**18**	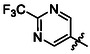	42 ± 10	>1.1	1.1 ± 0.11	27 ± 6
**19**	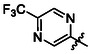	75 ± 8	–	–	–
**24**	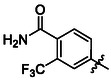	72 ± 2	>14	14 ± 1	32.5 ± 0.8
**25**	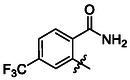	100	–	–	–
**38**	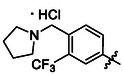	46 ± 2	>19	19 ± 1	<2.0
**39**	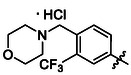	36 ± 7	>8.4	8.4 ± 1.1	4.2 ± 0.3
**40**	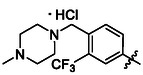	66 ± 10	>13	13 ± 0.8	43.5 ± 0.8
**41**	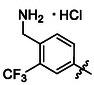	78 ± 5	–	–	–
**46**	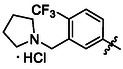	67 ± 7	>15	≈15	11.9 ± 0.4
**47**	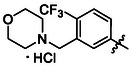	65 ± 5	>5.3	5.3 ± 0.48	<2.0
**48**	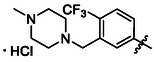	100	–	–	–

a) Residual activity (RA) in % at 10 μM concentration of screened compounds, SD standard deviation;

b) EC_50_ is a concentration required to reduce viral cytopathic effect by 50%;

c) CC_50_ is a concentration required to reduce cell viability by 50%, SE standard error of the mean.

**Table 3:** Residual activity (RA) of compounds with preserved 3‐(trifluoromethyl)phenyl ring, EC_50_ and CC_50_ values of selected compounds in Calu‐3 cells infected with SARS‐CoV‐2, and their kinetic solubility.

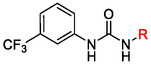						
Compound	R	RA ± SD [%]a)	EC_50_ [µM]b)	CC_50 _± SE [µM]c)		Kinetic solubility [µM]
**9**	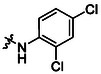	64 ± 12	>6.9	6.9 ± 0.94		<2.0
**10**	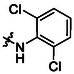	100	–	–		–
**28**	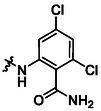	100	–	–		–
**54**	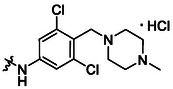	79 ± 5	–	–		–

a) Residual activity (RA) in % at 10 μM concentration of screened compounds, SD standard deviation;

b) EC_50_ is a concentration required to reduce viral cytopathic effect by 50%;

c) CC_50_ is a concentration required to reduce cell viability by 50%, SE standard error of the mean.

Substitution in the ortho position relative to the urea moiety, which induces distortion of molecular planarity, consistently resulted in diminished enzymatic activity. Compound **13** (RA = 34 ± 14%), with *p*‐CF_3_ group, was more potent than the parent compound **1** (RA = 45 ± 3%), following the trend previously observed for nitro‐substituted analogs in our earlier study.^[^ ^10^ ^]^ Interestingly, aza‐substitution at the *ortho* position (compounds **16**, **17**, and **19**) led to a marked decrease or complete loss of activity, whereas substitution at the meta position had minimal impact (compounds **15** and **18**). Introduction of a carboxamide group at any position on the aromatic ring similarly resulted in reduced activity (compounds **24**, **25**, and **28**).

Compounds bearing ionizable substituents revealed additional structure–activity relationship (SAR) trends. Compound **40** (RA = 66 ± 10%) exhibited higher potency than its regioisomer **54** (RA = 79 ± 5%), confirming that the RdRp binding pocket is asymmetric and accommodates bulkier substituents more favorably on the 3‐(trifluoromethyl)phenyl ring. Notably, whereas derivatives containing strong electron‐withdrawing groups (EWGs) in the *para* position were previously found to be 10–15% more potent than their *meta* analogs,^[^ ^10^ ^]^ compounds **46**–**48** and **38**–**40** displayed the opposite trend. This inversion may be attributed to steric hindrance, as the bulky solubilizing groups are less favorably accommodated in the *meta* position than in the *para* position. For compound **39** (RA = 36 ± 7%), the solubilizing moiety in the *para* position may form an additional interaction with the binding pocket—potentially via hydrogen bonding of the morpholine oxygen—resulting in enhanced activity compared to compound **1** (RA = 45 ± 3%).

Kinetic solubility measurements revealed that several derivatives exhibited improved solubility, exceeding 10 µM—the concentration used in the enzymatic assay—thus, validating the reliability of the biological data. For most compounds, enhanced solubility was associated with a reduction in enzymatic activity, with the notable exception of compound **18** (RA = 42 ± 10%), which maintained favorable levels in both parameters. Among derivatives with ionizable substituents, compounds **40** and **46** showed improved solubility, whereas **38** and **39** retained higher potency, suggesting that a balance between these two properties may be achievable within this scaffold.

Unfortunately, evaluation in the Calu‐3 cell‐based assay indicated that all tested compounds exhibited cytotoxicity at concentrations comparable to those required for antiviral efficacy (CC_50_ ≈ EC_50_; Table 2 and 3), underscoring the need for further structural optimization to improve the selectivity index.

### 2.3 Design of the Second Series of Compounds

Based on the structures and observed activities of compounds from the previous series (Table 2), we decided to perform trial molecular dynamics (MD) simulations with compounds **18**, **39**, and **47** in Pocket 1 identified in the previous article,^[^ ^10^ ^]^ but no stable position in the selected pocket was found.

**Figure 2:**
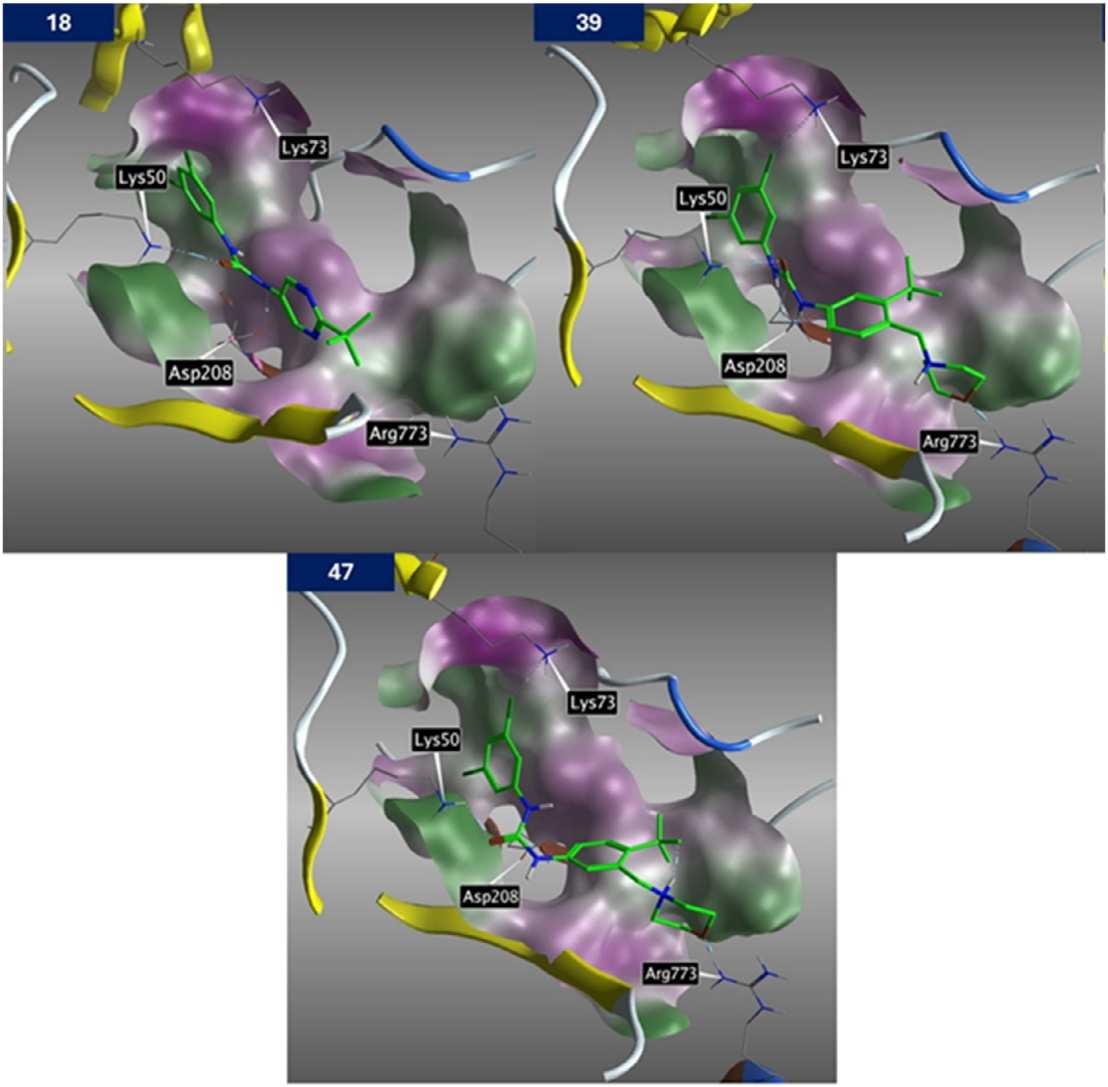
Molecular docking of compounds **18**, **39**, and **47** into the pocket of NiRAN domain of SARS‐CoV‐2 RdRp (PDB: 7ed5).

In the literature,^[^ ^13^ ^]^ the dual action of nucleoside inhibitor AT‐527 was reported, which is bound to both the main catalytic pocket and NiRAN domain of RdRp. Thus, molecular docking of compounds **18**, **39**, and **47** was performed into the pocket of NiRAN domain of SARS‐CoV‐2 RdRp complex (PDB: 7ed5), and it was found that they bind to the pocket in a similar manner (**Figure** **2**). Moreover, performed 200 ns MD simulation of **39** indicated that the molecule was stable in the selected pocket. There is more evidence supporting the binding of compound **39** (and other *N*,*N′*‐diphenylurea derivatives) to the pocket on the NiRAN domain. 1) The compounds potentiate the effect of remdesivir, suggesting they do not bind to the main catalytic pocket. 2) NiRAN has a guanylyl transferase function and, similarly to protein kinases, its pocket accommodates NTPs. Furthermore, protein kinase inhibitors often contain the *N*,*N′*‐diphenylurea scaffold; 3) The NiRAN pocket represents the most druggable pocket of the SARS‐CoV‐2 RdRp complex. 4) Compound **18** was found to be a competitive inhibitor of ATP in the performed enzymatic study and potentiates the effect of remdesivir. 5) The molecular docking showed that compound **39** binds to the NiRAN pocket better than compound **47** (Figure 2), which correlates with their activity on the enzymatic assay (Table 2).

The above evidence strongly supports the hypothesis that the actual binding site for our ligands is the NiRAN domain.

Docking results suggested that there are two main binding moieties of compound **39**, i.e., urea and morpholine (Figure 2). The phenyl rings seemed to fulfil a function as the space linker and the modulator of the acidity of the urea hydrogen atoms.

The oxygen of the morpholine moiety was shown to bind to protonated Arg773 as a hydrogen bond acceptor (HBA). This interaction can be improved by the implementation of a stronger HBA or by alteration of the linker geometry (improvement of the relative position of both HBA and arginine). These assumptions were the driving force for the design of the next series of compounds with improved aqueous solubility and binding to Arg773. Based on molecular docking and molecular modeling, 11 best scoring derivatives bearing a morpholine‐like moiety were selected.

### 2.4 Synthesis of the Second Series of Compounds

**Scheme 6:**
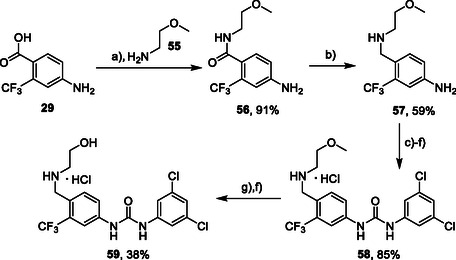
Synthesis of compounds **58** and **59**. Reagents and conditions: a) Et_3_N, HATU, DCM, RT; b) synhydride, THF, 50 °C; c) Boc_2_O, Et_3_N, THF, RT; d) **12**, DCM, 60 °C; e) TFA, RT; f) HCl, dioxane, RT; g) BBr_3_, DCM, RT.

Synthesis of compounds bearing 2‐methoxyethylamino and 2‐hydroxyethylamino moieties started with a reaction of compound **29** with 2‐methoxyethylamine (**55**) (**Scheme** **6**). The resulting amide **56** was reduced using synhydride to give amine **57**, which was protected on benzylic amine, treated with isocyanate **12**, and deprotected (in one pot) to give compound **58** in a good overall yield. Removal of the methyl ether group with BBr_3_ afforded compound **59** in a 38% yield.^[^ ^14^ ^]^

**Scheme 7:**
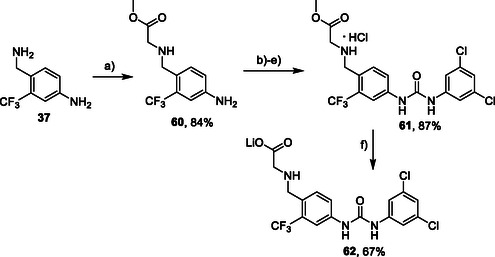
Synthesis of compounds **61** and **62**. Reagents and conditions: a) methyl bromoacetate, Et_3_N, THF, RT; b) Boc_2_O, Et_3_N, THF, RT; c) **12**, DCM, 60 °C; d) TFA, RT; e) HCl, dioxane, RT; f) LiOH.H_2_O, H_2_O/dioxane (1:1), RT.

Synthesis of the glycine analog **62** started with the alkylation of the commercially available benzylamine **37** with methyl bromoacetate (**Scheme** **7**). Intermediate **60** was protected on benzylic amine, treated with isocyanate **12**, and deprotected (in one pot) to give compound **61**. Subsequent hydrolysis of the methyl ester afforded compound **62** as lithium salt.

**Scheme 8:**
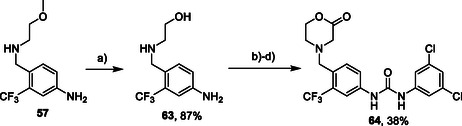
Synthesis of compound **64**. Reagents and conditions: a) BBr_3_, DCM, RT; b) methyl bromoacetate, Et_3_N, THF, RT; c) PTSA, RT; d) **12**, Et_3_N, DCM, 60 °C.

Compounds with modified morpholine moiety were prepared by two routes. Synthesis of compound **64** started from intermediate **57** by removal of the methyl ether group using BBr_3_ (**Scheme** **8**). Alcohol **63** was alkylated with methyl bromoacetate and the reaction mixture was acidified with PTSA to afford crude lactone intermediate.^[^ ^15^ ^]^ An excess of trimethylamine was added to the reaction mixture, followed by isocyanate **12** to give compound **64**.

**Scheme 9:**
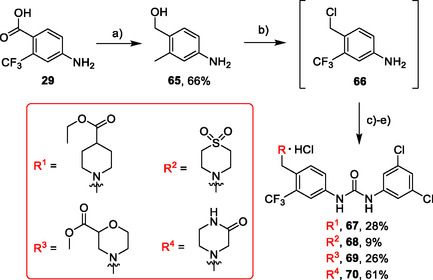
Synthesis of compounds **67–70**. Reagents and conditions: a) synhydride, THF, 50 °C; b) SOCl_2_, 70 °C; c) secondary amine, DCM, DBU, RT; d) **12**, DCM, 60 °C; e) HCl, dioxane, RT.

Another synthetic approach exploited a reduction of **29** with synhydride to alcohol **65**, followed by transformation of the alcohol into chloride and reaction with a secondary amines. Chloride intermediate appeared to be extremely reactive and was added dropwise to the excess of the secondary amine because, otherwise, a formation of quaternary ammonium salts as a product of DBU alkylation was observed in UPLC‐MS spectra of the reaction mixtures. Tertiary amines reacted with isocyanate **12** to give compounds **67–70** as HCl salts (**Scheme** **9**).

**Scheme 10:**
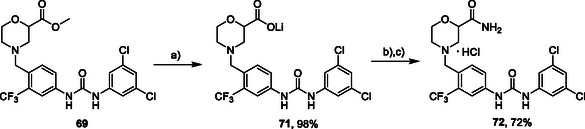
Synthesis of compounds **71** and **72**: Reagents and conditions: a) LiOH.H_2_O, H_2_O/dioxane (1:1), RT; b) NH_4_Cl, DBU, HATU, DCM, RT; c) HCl, dioxane, RT.

Synthesis of compound **71** was performed via hydrolysis of compound **69** under mild conditions. In the following step, **71** was transformed into amide **72** via coupling with HATU (**Scheme** **10**).

### 2.5 Biological Activity of the Second Series of Compounds

Several analogs of compound **39** (RA = 36 ± 7%) exhibited similar or slightly lower activities (**Table** **4**). Based on the activity in enzymatic assay, additional data were collected for selected compounds, i.e., cell‐based assay activity, toxicity, and kinetic solubility.

**Table 4:** Residual activity (RA) of tested compounds and EC_50_ and CC_50_ values of selected compounds in Calu‐3 cells infected with SARS‐CoV‐2, and their kinetic solubility.

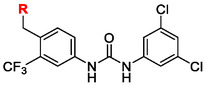							
Compound		R	RA ± SD [%]a)	EC_50_ [µM]b)		CC_50_ ± SE [µM]c)	Kinetic solubility [µM]
**58**		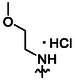	100	–		–	–
**59**		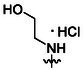	76 ± 11	–		–	–
**61**		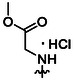	43 ± 8	>8.4		8.4 ± 0.46	7.0 ± 0.6
**62**		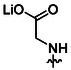	70 ± 10	–		–	–
**64**		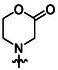	39 ± 12	>38		38 ± 8.2	21 ± 2
**67**		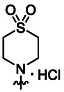	51 ± 11	>9		9.0 ± 0.38	<2,0
**68**		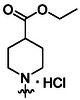	37 ± 5	>5.9		5.9 ± 0.51	2.57 ± 0.04
**69**		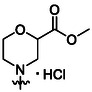	42 ± 4	>34		34 ± 5.6	<2,0
**70**		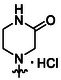	63 ± 7	>9.5		9.5 ± 0.28	11.20 ± 0.07
**71**		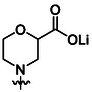	63 ± 7	>32		32 ± 0.77	71 ± 1
**72**		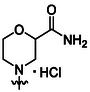	52 ± 5	>12		12 ± 0.51	4.7 ± 0.1

a) Residual activity (RA) in % at 10 μM concentration of screened compounds, SD standard deviation;

b) EC_50_ is a concentration required to reduce viral cytopathic effect by 50%;

c) CC_50_ is a concentration required to reduce cell viability by 50%, SE standard error of the mean.

This series further supports the positive influence of a strong hydrogen‐bond acceptor (HBA) within the solubilizing moiety of the ligand, as illustrated by several representative examples. Compound **39** (RA = 36 ± 7%) displayed markedly higher activity than compound **58** (RA = 100%) despite their overall structural similarity. This difference can be attributed to the presence of a well‐oriented HBA in the morpholine ring of compound **39**, which enables directed hydrogen bonding, whereas the methoxy group in compound **58**—positioned on a flexible linker—is less capable of forming such an interaction. A comparable trend was observed between compounds **58** (RA = 100%) and **61** (RA = 43 ± 8%), both featuring flexible solubilizing linkers but differing in their polar functionalities: the methyl carboxylate group of **61** serves as a substantially stronger HBA than the methoxy group of **58**, correlating with enhanced inhibitory activity.

Consistent with these observations, amide derivatives exhibited lower activity than the corresponding esters, reflecting the weaker HBA character of the amide moiety. This trend is evident in compound pairs **64**/**70** and **69**/**72** (Table 4).

Biological evaluation in cell‐based assays revealed no significant improvement in cytotoxicity relative to the preceding compound series. Compounds **61** and **64** (Table 4) demonstrated increased kinetic solubility (1.7‐fold and 5‐fold higher, respectively) compared with compound **39** (Table 2). However, compound **64** was found to be chemically unstable and underwent rapid hydrolysis in aqueous solution, casting doubt on the accuracy of its solubility measurements. Furthermore, hydrolysis resulted in the complete loss of activity, rendering this compound unsuitable for further development. In contrast, compound **61** exhibited a favorable balance between potency and solubility, representing a promising lead within this subset of compounds. Compound **71** achieved the highest solubility in the series but displayed comparatively low enzymatic activity (Table 4).

## 3 Conclusions

A series of compounds was synthesized and evaluated in an enzymatic assay assessing their residual activity (RA) of the SARS‐CoV‐2 RNA‐dependent RNA polymerase (RdRp) complex (RA is the residual activity at 10 μM concentration of a compound calculated as the area under the amplification curve (AUC) of the reaction in the presence of the compound divided by the AUC of the noninhibited reaction). Structural modifications aimed at improving aqueous solubility were systematically explored. The introduction of solubilizing aliphatic amino groups (as hydrochloride salts) or the substitution of an aromatic ring with a heteroaromatic moiety emerged as the most effective strategies. Among the synthesized derivatives, compounds **18** (RA = 42 ± 10%; kinetic solubility = 27 µM/L), **39** (RA = 36 ± 7%; 4.2 µM L^−1^), and **61** (RA = 43 ± 12%; 21 µM L^−1^) exhibited the most favorable balance between potency and solubility.

In addition, several analogs bearing solubilizing substituents demonstrated markedly improved aqueous solubility—namely compounds **24** (32.5 µM L^−1^), **40** (43.5 µM L^−1^), and **71** (71 µM L^−1^)—albeit with reduced enzymatic inhibition (all with RA around 65%). Although all tested compounds displayed notable cytotoxicity in the Calu‐3 cell‐based assay, these results provide valuable insights into the rational design of allosteric SARS‐CoV‐2 RdRp inhibitors. Ongoing optimization efforts are focused on enhancing their pharmacological profile while mitigating cytotoxic effects.

## 4 Experimental Section

4.1

4.1.1

##### General Methods

Unless otherwise stated, solvents were evaporated at 40 °C/2 kPa and prepared compounds were dried at 30 °C at 2 kPa. Starting compounds and reagents were purchased from commercial suppliers (Sigma–Aldrich, Fluorochem, Acros Organics, TCI, AmBeed, and Vitas‐M Laboratory) and used without further purification or were prepared according to the published procedures. Tetrahydrofuran (THF), dioxane, and acetonitrile were dried by activated neutral alumina (drysphere). Dimethylformamide (DMF) was dried by activated molecular sieves (3 Å). Other dry solvents were purchased from commercial suppliers (Sigma–Aldrich and Acros Organics). Triethylamine was dried over potassium hydroxide under an argon atmosphere in dark flask sealed with septum.

Flash column chromatography was carried out by Teledyne ISCO Grace with dual absorbance detector on Teledyne ISCO columns RediSepRf HP C18 Aq GOLD in sizes 50 and 100 g. Eluents used were methanol, acetonitrile, and water. Preparative LC purifications were performed on Waters Delta 600 chromatography system with columns packed with C18 reversed phase resin—Phenomenex Gemini 10 μm 21 × 250 mm. Mass spectra, UV absorbance, and purity of compounds were measured on Waters UPLC‐MS system consisting of Waters UPLC H‐Class Core System (column Waters Cortecs UPLC C18 1.6 µm, 2.1 × 50 mm), Waters Acquity UPLC PDA detector, and Mass spectrometer Waters SQD2. Yields were determined based on the amount of isolated compound. All final compounds were >95% pure by HPLC analysis. The universal LC method was used (eluent H_2_O/CH_3_CN with 0.1% of formic acid in both mobile phases, gradient 0–100%, run length 3.5 min) and MS method (ESI + and/or ESI‐ cone voltage = 30 V, mass detector range 100–1000 Da). NMR spectra were recorded on Bruker Avance III HD spectrometers (^1^H at 400, 500 or 600 MHz) in DMSO‐*d*_6_, CDCl_3_ or D_2_O using a solvent signal as a reference (DMSO‐*d*_6_: 2.50 and 39.52, CDCl_3_: 7.26 and 77.00 for ^1^H and ^13^C, respectively). All structures were confirmed, and ^1^H and ^13^C signals were assigned by combining 1D and 2D NMR (H, C‐HSQC, H, C‐HMBC) experiments. High‐resolution mass spectra were measured on a LTQ Orbitrap XL spectrometer (Thermo Fisher Scientific).

##### General Procedure for the Synthesis of Isocyanates (Method A)

A mixture of corresponding aniline (18.5 mmol, 1.0 equiv) in DCM (30 mL) and Et_3_N (7.7 mL, 55.5 mmol, 3.0 equiv) was added dropwise (over 40 min) to the ice‐cold solution of triphosgene (5.5 g, 18.5 mmol, 1.0 equiv) in DCM (30 mL), and the resulting mixture was stirred for 30 min. The solvent was removed under reduced pressure and the residue was used in the following reaction without further purification (the reaction conversion and the product content were estimated using UPLC‐MS).

##### General Procedure for the synthesis of Substituted N, N′‐Diphenylurea Derivatives via Coupling of Anilines with Isocyanates (Method B)

Corresponding aniline (1.06 mmol, 1.0 equiv) was dissolved in DCM (10 mL), and the crude isocyanate mixture was added (estimated 1.0 equiv). The reaction mixture was heated to 60 °C for 12 h. The solvent was removed under reduced pressure, and the residue was dissolved in DMF, applied on flash chromatography column, and separated (RP ‐ C18aq, eluent water/methanol, gradient 0‐100%) to obtain the final product.

##### General Procedure for the Synthesis of Benzoic Acid Aliphatic Amides (Method C)

A liphatic amine (1.94 mmol, 3.0 equiv), Et_3_N (0.2 mL, 1.46 mmol, 3.0 equiv), and after 5 min HATU (240 mg, 0.63 mmol, 1.3 equiv) were added to the mixture of the corresponding benzoic acid (0.50 mmol, 1.0 equiv) in DCM (5.0 mL). The reaction mixture was stirred for 1 h at RT, the solvent was removed under reduced pressure, and the residue was dissolved in DMF, applied on flash chromatography column, and separated (RP ‐ C18aq, eluent water/methanol, gradient 0‐100%) to obtain the final product.

##### General Procedure for the Reduction of Benzoic Acid Aliphatic Amides to Aliphatic Amines (Method D)

Synhydrid (0.2 mL of 3.5 M solution in toluene, 0.73 mmol, 8.0 equiv) was added dropwise to a solution of the corresponding aliphatic amide (0.10 mmol, 1.0 equiv) in dry THF (5.0 mL) at 65 °C. The reaction mixture was stirred for 1 h at RT, cooled down to 0 °C, and 1 M aqueous solution of NaOH was added, followed by the extraction with DCM (3 × 10 mL). Combined organic fractions were evaporated, and the residue dissolved in DMF, applied on flash chromatography column, and separated (RP ‐ C18aq, eluent water/methanol, gradient 0‐100%) to obtain the aliphatic amine.

##### General Procedure for Converting Free Amines into Hydrochlorides (Method E):

Several drops of 4 M HCl in dioxane were added to a solution of free amine (1.0 equiv.) in dry dioxane (5 mL). The formed suspension was centrifuged, the liquid was removed, and the solid residue was dried under reduced pressure to give the desired hydrochloride salt.

##### 1‐Isocyanato‐3‐(Trifluoromethyl)Benzene (6)


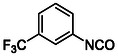


3‐(Trifluoromethyl)aniline (2.5 g, 15.5 mmol, 1.0 eq) was treated according to Method A. The resulting crude product **6** (9.4 g, 28% content of the product) was used without further purifications in the next reactions.

##### 1‐(2,4‐Dichlorophenyl)‐3‐(3‐(Trifluoromethyl)Phenyl)Urea (9)


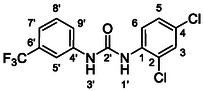


Compound **6** (232 mg of the crude mixture, 0.34 mmol, 1.0 eq) and 2,4‐dichloroaniline (56 mg, 0.34 mmol, 1.0 eq) were treated according to Method B to give **9** (100 mg, 83%) as a white solid. ^1^H NMR (DMSO, 401 MHz) *δ* 9.77 (1H, s, H‐3), 8.46 (1H, s, H‐1^′^), 8.18 (1H, d, *J* = 9.0 Hz, H‐6), 8.05—8.01 (1H, m, H‐5^′^), 7.64 (1H, d, *J* = 2.5 Hz, H‐3), 7.56—7.53 (2H, m, H‐8^′^, H‐9^′^), 7.39 (1H, dd, *J* = 9.0, 2.5 Hz, H‐5), 7.36—7.32 (1H, m, H‐7^′^). ^13^C NMR (DMSO, 101 MHz) *δ* 152.03 (C‐2′), 140.12 (C‐4^′^), 134.88 (C‐1), 130.11 (C‐8^′^), 129.63 (q, *J* = 31.5 Hz, C‐6^′^), 128.62 (C‐3), 127.69 (C‐5), 126.57 (C‐4), 124.16 (q, *J* = 272.2 Hz, CF_3_), 123.05 (C‐2), 122.48 (C‐6), 121.84 (C‐9^′^), 118.55 (q, *J* = 4.0 Hz, C‐7^′^), 114.12 (q, *J* = 4.1 Hz, C‐5^′^). HRMS (ESI+): *m/z* [M + H]^+^ calculated for C_14_H_10_ON_2_Cl_2_F_3_ = 349.0117, found: 349.0120.

##### 1‐(2,6‐Dichlorophenyl)‐3‐(3‐(Trifluoromethyl)Phenyl)Urea (10)


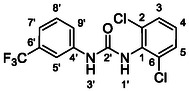


Compound **6** (232 mg of the crude mixture, 0.34 mmol, 1.0 eq) and 2,4‐dichloroaniline (56 mg, 0.34 mmol, 1.0 eq) were treated according to Method B to give **10** (42 mg, 35%) as a white solid. ^1^H NMR (DMSO, 401 MHz) *δ* 9.62 (1H, s, H‐3^′^), 8.52 (1H, s, H‐1^′^), 8.00 (1H, dd, *J* = 2.1, 2.1 Hz, H‐5^′^), 7.64—7.60 (1H, m, H‐9^′^), 7.54 (2H, d, *J* = 8.1 Hz, H‐3, H‐5), 7.50 (1H, dd, *J* = 8.0, 8.0 Hz, H‐8^′^), 7.35—7.31 (1H, m, H‐4), 7.31—7.28 (1H, m, H‐7^′^). ^13^C NMR (DMSO, 101 MHz) *δ* 152.49 (C‐2′), 140.74 (C‐4^′^), 134.11 (C‐2, C‐6), 133.02 (C‐1), 129.91 (C‐8^′^), 129.49 (q, *J* = 31.5 Hz, C‐6^′^), 128.68 (C‐4), 128.48 (C‐3, C‐5), 124.21 (q, *J* = 272.5 Hz, CF_3_), 121.61 (C‐9^′^), 118.03 (q, *J* = 4.2 Hz, C‐7^′^), 113.92 (q, *J* = 4.2 Hz, C‐5^′^). HRMS (ESI+): *m/z* [M + H]^+^ calculated for C_14_H_10_ON_2_Cl_2_F_3_ = 349.0117, found: 349.0120.

##### 1‐Isocyanato‐3,5‐Dichlorobenzene (12)


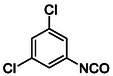


3,5‐Dichloroaniline (3.0 g, 18.5 mmol, 1.0 eq) was treated according to Method A. The resulting crude product **12** (10.9 g, 32% content of the product) was used without further purifications in the next reactions.

##### 1‐(3,5‐Dichlorophenyl)‐3‐(4‐(Trifluoromethyl)Phenyl)Urea (13)


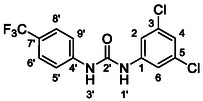


Compound **12** (625 mg of the crude mixture) 1.10 mmol, 1.0 eq) and 4‐(trifluoromethyl)aniline (171 mg, 1.10 mmol, 1.0 eq) were treated according to Method B to give **13** (305 mg, 82%) as a brownish solid. ^1^H NMR (DMSO, 401 MHz) *δ* 9.34 (1H, s, H‐3^′^), 9.22 (1H, s, H‐1^′^), 7.69—7.62 (4H, m, H‐5^′^, H‐6^′^, H‐8^′^, H‐9^′^), 7.54 (2H, d, *J* = 1.9 Hz, H‐2, H‐6), 7.19 (1H, t, *J* = 1.9 Hz, H‐4). ^13^C NMR (DMSO, 101 MHz) *δ* 152.05 (C‐2′), 142.96 (C‐4^′^), 141.91 (C‐1), 134.10 (C‐3, C‐5), 126.08 (q, *J* = 3.9 Hz, C‐6^′^, C‐8^′^), 124.48 (q, *J* = 271.4 Hz, CF_3_), 122.23 (q, *J* = 31.9 Hz, C‐7^′^), 121.26 (C‐4), 118.23 (C‐5^′^, C‐9^′^), 116.55 (C‐2, C‐6). HRMS (ESI+): *m/z* [M + H]^+^ calculated for C_14_H_10_ON_2_Cl_2_F_3_ = 349.0117, found: 349.0119.

##### 1‐(3,5‐Dichlorophenyl)‐3‐(2‐(Trifluoromethyl)Phenyl)Urea (14)


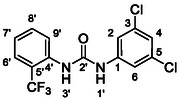


Compound **12** (625 mg of the crude mixture, 1.10 mmol, 1.0 eq) and 2‐(trifluoromethyl)aniline (171 mg, 1.10 mmol, 1.0 eq) were treated according to Method B to give **14** (285 mg, 77%) as a white solid. ^1^H NMR (DMSO, 401 MHz) *δ* 9.72 (1H, s, H‐3^′^), 8.24 (1H, s, H‐1^′^), 7.87 (1H, d, *J* = 8.2 Hz, H‐9^′^), 7.70 (1H, dd, *J* = 8.0, 1.5 Hz, H‐6^′^), 7.65 (1H, ddd, *J* = 7.9, 7.8, 1.5 Hz, H‐8^′^), 7.52 (2H, d, *J* = 1.9 Hz, H‐2, H‐6), 7.37—7.30 (1H, m, H‐7^′^), 7.18 (1H, t, *J* = 1.9 Hz, H‐4). ^13^C NMR (DMSO, 101 MHz) *δ* 152.41 (C‐2′), 142.01 (C‐1), 135.67 (q, *J* = 2.0 Hz, C‐4^′^), 134.16 (C‐3, C‐5), 132.95 (C‐8^′^), 126.51 (C‐9^′^), 126.01 (q, *J* = 5.3 Hz, C‐6^′^), 124.51 (C‐7^′^), 123.87 (q, *J* = 272.9 Hz, CF_3_), 121.18 (C‐4), 120.92 (q, *J* = 29.2 Hz, C‐5^′^), 116.28 (C‐2, C‐6). HRMS (ESI+): *m/z* [M + H]^+^ calculated for C_14_H_10_ON_2_Cl_2_F_3_ = 349.0117, found: 349.0119.

##### 1‐(3,5‐Dichlorophenyl)‐3‐(6‐(Trifluoromethyl)Pyridin‐3‐Yl)Urea (15)


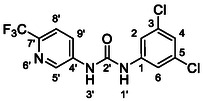


Compound **12** (313 mg of the crude mixture, 0.53 mmol, 1.0 eq) and 5‐(trifluoromethyl)pyridin‐2‐amine (86 mg, 0.53 mmol, 1.0 eq) were treated according to Method B to give **15** (160 mg, 86%) as a white solid. ^1^H NMR (DMSO, 401 MHz) *δ* 9.51 (1H, s, H‐3^′^), 9.32 (1H, s, H‐1^′^), 8.76 (1H, d, *J* = 2.5 Hz, H‐5^′^), 8.19 (1H, dd, *J* = 8.9, 2.4 Hz, H‐9^′^), 7.82 (1H, d, *J* = 8.7 Hz, H‐8^′^), 7.55 (2H, d, *J* = 1.9 Hz, H‐2, H‐6), 7.19 (1H, t, *J* = 1.9 Hz, H‐4). ^13^C NMR (DMSO, 101 MHz) *δ* 152.09 (C‐2′), 141.66 (C‐1), 140.18 (C‐5^′^), 139.49 (q, *J* = 34.2 Hz, C‐7^′^), 139.06 (C‐4^′^), 134.12 (C‐3, C‐5), 125.69 (C‐9^′^), 121.87 (q, *J* = 272.9 Hz, CF_3_), 121.54 (C‐4), 121.14 (q, *J* = 2.9 Hz, C‐8^′^), 116.77 (C‐2, C‐6). HRMS (ESI+): *m/z* [M + H]^+^ calculated for C_13_H_9_ON_3_Cl_2_F_3_ = 350.0069, found: 350.0070.

##### 1‐(3,5‐Dichlorophenyl)‐3‐(5‐(Trifluoromethyl)Pyridin‐2‐Yl)Urea (16)


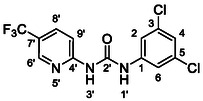


Compound **12** (313 mg of the crude mixture, 0.53 mmol, 1.0 eq) and 5‐(trifluoromethyl)pyridin‐2‐amine (86 mg, 0.53 mmol, 1.0 eq) were treated according to Method B to give **16** (35 mg, 19%) as a yellowish solid. ^1^H NMR (DMSO, 401 MHz) *δ* 10.30 (2H, s, H‐1^′^, H‐3^′^), 8.70 (1H, d, *J* = 2.5 Hz, H‐6^′^), 8.14 (1H, dd, *J* = 8.9, 2.6 Hz, H‐8^′^), 7.73 (1H, d, *J* = 8.8 Hz, H‐9^′^), 7.64 (2H, d, *J* = 1.9 Hz, H‐2, H‐6), 7.24 (1H, t, *J* = 1.9 Hz, H‐4). ^13^C NMR (DMSO, 101 MHz) *δ* 155.33 (C‐2′), 151.66 (C‐4^′^), 144.83 (q, *J* = 4.3 Hz, C‐6^′^), 141.20 (C‐1), 135.95 (q, *J* = 3.7 Hz, C‐8^′^), 134.21 (C‐3, C‐5), 123.99 (q, *J* = 271.1 Hz, CF_3_), 121.95 (C‐4), 118.94 (q, *J* = 32.7 Hz, C‐7^′^), 117.06 (C‐2, C‐6), 111.84 (C‐9^′^). HRMS (ESI+): *m/z* [M + H]^+^ calculated for C_13_H_9_ON_3_Cl_2_F_3_ = 350.0069, found: 350.0070.

##### 1‐(3,5‐Dichlorophenyl)‐3‐(6‐(Trifluoromethyl)Pyridazin‐3‐Yl)Urea (17)


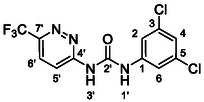


Compound **12** (156 mg of the crude mixture, 0.27 mmol, 1.0 eq) and 6‐(trifluoromethyl)pyridazin‐3‐amine (43 mg, 0.27 mmol, 1.0 eq) were treated according to Method B to give **17** (43 mg, 46%) as a reddish solid. ^1^H NMR (DMSO, 401 MHz) *δ* 10.44 (1H, s, H‐3^′^), 9.71 (1H, s, H‐1^′^), 8.34 (1H, dd, *J* = 9.4, 0.7 Hz, H‐5^′^), 8.18 (1H, d, *J* = 9.4 Hz, H‐6^′^), 7.58 (2H, d, *J* = 1.9 Hz, H‐2, H‐6), 7.28 (1H, t, *J* = 1.9 Hz, H‐4). ^13^C NMR (DMSO, 101 MHz) *δ* 157.72 (C‐2′), 151.57 (C‐4^′^), 146.04 (q, *J* = 34.1 Hz, C‐7^′^), 140.97 (C‐1), 134.27 (C‐3, C‐5), 126.50 (q, *J* = 2.6 Hz, C‐6^′^), 122.23 (C‐4), 117.79 (C‐5^′^), 117.10 (C‐2, C‐6). HRMS (ESI+): *m/z* [M + H]^+^ calculated for C_12_H_8_ON_4_Cl_2_F_3_ = 351.0022, found: 351.0025.

##### 1‐(3,5‐Dichlorophenyl)‐3‐(2‐(Trifluoromethyl)Pyrimid‐5‐Yl)Urea (18)


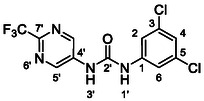


Compound **12** (156 mg of the crude mixture, 0.27 mmol, 1.0 eq) and 2‐(trifluoromethyl)pyrimidin‐5‐amine (43 mg, 0.27 mmol, 1.0 eq) were treated according to Method B to give **18** (92 mg, 99%) as a white solid. ^1^H NMR (DMSO, 401 MHz) *δ* 9.62 (2H, s, H‐1^′^, H‐3^′^), 9.11 (2H, s, H‐5^′^), 7.57 (2H, d, *J* = 1.9 Hz, H‐2, H‐6), 7.23 (1H, t, *J* = 1.9 Hz, H‐4). ^13^C NMR (DMSO, 101 MHz) *δ* 151.95 (C‐2′), 148.05 (q, *J* = 36.1 Hz, C‐7^′^), 146.88 (C‐5^′^), 141.46 (C‐1), 136.88 (C‐4^′^), 134.16 (C‐3, C‐5), 121.82 (C‐4), 119.81 (q, *J* = 274.1 Hz, CF_3_), 116.94 (C‐2, C‐6). HRMS (ESI+): *m/z* [M + H]^+^ calculated for C_12_H_8_ON_4_Cl_2_F_3_ = 351.0022, found: 351.0025.

##### 1‐(3,5‐Dichlorophenyl)‐3‐(5‐(Trifluoromethyl)Pyrazin‐2‐Yl)Urea (19)


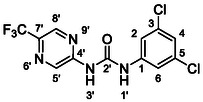


Compound **12** (156 mg of the crude mixture, 0.27 mmol, 1.0 eq) and 5‐(trifluoromethyl)pyrazin‐2‐amine (43 mg, 0.27 mmol, 1.0 eq) were treated according to Method B to give **19** (33 mg, 35%) as a white solid. ^1^H NMR (DMSO, 401 MHz) *δ* 10.30 (1H, s, H‐3^′^), 9.97 (1H, s, H‐1^′^), 9.13 (1H, d, *J* = 1.5 Hz, H‐5^′^), 8.83 (1H, d, *J* = 0.8 Hz, H‐8^′^), 7.61 (2H, d, *J* = 1.9 Hz, H‐2, H‐6), 7.27 (1H, t, *J* = 1.9 Hz, H‐4). ^13^C NMR (DMSO, 101 MHz) *δ* 151.43 (C‐4^′^), 151.25 (C‐2′), 140.90 (C‐1), 139.56 (q, *J* = 3.5 Hz, C‐8^′^), 135.05 (C‐5^′^), 135.03 (q, *J* = 34.9 Hz, C‐7^′^), 134.27 (C‐3, C‐5), 122.28 (C‐4), 121.71 (q, *J* = 272.9 Hz, CF_3_), 117.16 (C‐2, C‐6). HRMS (ESI+): *m/z* [M + H]^+^ calculated for C_12_H_8_ON_4_Cl_2_F_3_ = 351.0022, found: 351.0024.

##### 4‐Amino‐2‐(Trifluoromethyl)Benzamide (22)


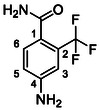


Compound **26** (1.50 g, 7.3 mmol, 1.0 eq), ammonium chloride (0.78 g, 14.6 mmol, 2.0 eq), Et_3_N (2.0 mL, 14.6 mmol, 2.0 eq), and HATU (3.06 g, 8.04 mmol, 1.1 eq) were treated according to Method C to give **22** (0.50 g, 34%) as a white solid. ^1^H NMR (DMSO, 401 MHz) *δ* 7.57 (1H, s, CON**H**_2_a), 7.23 (1H, d, *J* = 8.3 Hz, H‐6), 7.16 (1H, s, CON**H**_2_b), 6.88 (1H, d, *J* = 2.2 Hz, H‐3), 6.72 (1H, dd, *J* = 8.3, 2.3 Hz, H‐5), 5.78 (2H, s, C4‐N**H**_2_). ^13^C NMR (DMSO, 101 MHz) *δ* 169.48 (CON), 149.94 (C‐4), 129.96 (C‐6), 127.29 (q, *J* = 30.5 Hz, C‐2), 124.01 (q, *J* = 273.6 Hz, CF_3_), 123.10 (q, *J* = 2.1 Hz, C‐1), 115.33 (C‐5), 110.62 (q, *J* = 5.4 Hz, C‐3). MS (ESI+): *m/z* [M + H]^+^ calculated for C_8_H_8_ON_2_F_3_ = 205.1, found: 205.2.

##### 2‐Amino‐4‐(Trifluoromethyl)Benzamide (23)


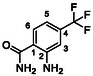


2‐Amino‐4‐trifluoromethylbenzoic acid (200 mg, 0.98 mmol, 1.0 eq), ammonium chloride (209 mg, 3.90 mmol, 4.0 eq), Et_3_N (0.41 mL, 2.93 mmol, 3.0 eq), and HATU (482 mg, 1.27 mmol, 1.3 eq) were treated according to Method C to give **23** (170 mg, 85%) as a white solid. ^1^H NMR (DMSO, 401 MHz) *δ* 7.93 (1H, s, CON**H**_2_a), 7.73—7.65 (1H, m, H‐6), 7.33 (1H, s, CON**H**_2_b), 7.03 (1H, d, *J* = 1.9 Hz, H‐3), 6.87 (2H, s, C2‐N**H**_2_), 6.75 (1H, dd, *J* = 8.3, 1.9 Hz, H‐5). ^13^C NMR (DMSO, 101 MHz) *δ* 170.20 (CON), 150.20 (C‐2), 131.81 (q, *J* = 31.3 Hz, C‐4), 129.89 (C‐6), 124.00 (q, *J* = 272.5 Hz, CF_3_), 116.74 (C‐1), 112.51 (q, *J* = 4.1 Hz, C‐3), 109.91 (q, *J* = 3.7 Hz, C‐5). MS (ESI+): *m/z* [M + H]^+^ calculated for C_8_H_8_ON_2_F_3_ = 205.1, found: 205.2.

##### 4‐(3‐(3,5‐Dichlorophenyl)Ureido)‐2‐(Trifluoromethyl)Benzamide (24)


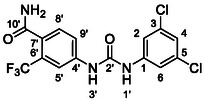


Compound **12** (156 mg of the crude mixture, 0.27 mmol, 1.0 eq) and **22** (54 mg, 0.27 mmol, 1.0 eq) were treated according to Method B to give **24** (93 mg, 89%) as a yellowish solid. ^1^H NMR (DMSO, 401 MHz) *δ* 9.38 (1H, s, H‐3^′^), 9.24 (1H, s, H‐1^′^), 7.98 (1H, d, *J* = 2.1 Hz, H‐5^′^), 7.85 (1H, s, CON**H**_2_a), 7.64 (1H, dd, *J* = 8.4, 2.2 Hz, H‐9^′^), 7.55 (2H, d, *J* = 1.9 Hz, H‐2, H‐6), 7.51—7.46 (2H, m, CON**H**_2_b, H‐8^′^), 7.20 (1H, t, *J* = 1.8 Hz, H‐4). ^13^C NMR (DMSO, 101 MHz) *δ* 168.81 (C‐10’), 152.20 (C‐2′), 141.89 (C‐1), 140.31 (C‐4^′^), 134.11 (C‐3, C‐5), 130.32 (C‐7^′^), 129.37 (C‐8^′^), 126.50 (q, *J* = 31.3 Hz, C‐6^′^), 123.63 (q, *J* = 274.0 Hz, CF_3_), 121.34 (C‐4), 121.21 (C‐9^′^), 116.69 (C‐2, C‐6), 115.54 (q, *J* = 5.6 Hz, C‐5^′^). HRMS (ESI+): *m/z* [M + H]^+^ calculated for C_15_H_11_O_2_N_3_Cl_2_F_3_ = 392.0175, found: 392.0176.

##### 2‐(3‐(3,5‐Dichlorophenyl)Ureido)‐4‐(Trifluoromethyl)Benzamide (25)


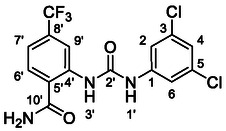


Compound **12** (156 mg of the crude mixture, 0.27 mmol, 1.0 eq) and **23** (54 mg, 0.27 mmol, 1.0 eq) were treated according to Method B to give **25** (65 mg, 62%) as a white solid. ^1^H NMR (DMSO, 401 MHz) *δ* 10.82 (1H, s, H‐3^′^), 10.26 (1H, s, H‐1^′^), 8.68 (1H, d, *J* = 1.9 Hz, H‐9^′^), 8.45 (1H, s, CON**H**_2_a), 7.95 (1H, s, CON**H**_2_b), 7.92 (1H, d, *J* = 7.8 Hz, H‐6^′^), 7.59 (2H, d, *J* = 1.9 Hz, H‐2, H‐6), 7.41 (1H, dd, *J* = 8.2, 1.2 Hz, H‐7^′^), 7.18 (1H, t, *J* = 1.9 Hz, H‐4). ^13^C NMR (DMSO, 101 MHz) *δ* 169.55 (C‐10’), 152.02 (C‐2′), 142.15 (C‐1), 140.34 (C‐4^′^), 134.03 (C‐3, C‐5), 131.51 (q, *J* = 31.8 Hz, C‐8^′^), 129.55 (C‐6^′^), 123.72 (q, *J* = 272.7 Hz, CF_3_), 123.27 (C‐5^′^), 121.24 (C‐4), 117.53 (C‐7^′^), 116.62 (C‐2, C‐6), 116.40 (q, *J* = 4.3 Hz, C‐9^′^). HRMS (ESI+): *m/z* [M + H]^+^ calculated for C_15_H_11_O_2_N_3_Cl_2_F_3_ = 392.0175, found: 392.0176.

##### 2‐Amino‐4,6‐Dichlorobenzamide (27)


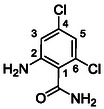


2‐Amino‐4,6‐dichlorobenzoic acid (100 mg, 0.49 mmol, 1.0 eq), ammonium chloride (104 mg, 1.94 mmol, 4.0 eq), Et_3_N (0.20 mL, 1.46 mmol, 3.0 eq), and HATU (240 mg, 0.63 mmol, 1.3 eq) were treated according to Method C to give **27** (70 mg, 70%) as a yellowish oil. ^1^H NMR (DMSO, 401 MHz) *δ* 7.88 (1H, s, CON**H**_2_a), 7.66 (1H, s, CON**H**_2_b), 6.73 (1H, d, *J* = 1.9 Hz, H‐3), 6.67 (1H, d, *J* = 1.9 Hz, H‐5). ^13^C NMR (DMSO, 101 MHz) *δ* 166.63 (CON), 147.69 (C‐2), 133.60 (C‐4), 131.10 (C‐6), 120.78 (C‐1), 115.41 (C‐5), 113.05 (C‐3). MS (ESI+): *m/z* [M + H]^+^ calculated for C_7_H_7_ON_2_Cl_2_ = 205.0, found: 205.2.

##### 2,4‐Dichloro‐6‐(3‐(3‐(Trifluoromethyl)Phenyl)Ureido)Benzamide (28)


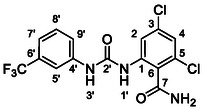


Compound **6** (50 mg (179 mg of crude mixture), 0.27 mmol, 1.0 eq) and **27** (55 mg, 0.27 mmol, 1.0 eq) were treated according to Method B to give **28** (12 mg, 12%) as a white solid. ^1^H NMR (DMSO, 401 MHz) *δ* 7.83—7.79 (2H, m, H‐5^′^, H‐7^′^), 7.77—7.70 (1H, m, H‐8^′^), 7.68—7.65 (1H, m, H‐10’), 7.42 (1H, d, *J* = 2.0 Hz, H‐4), 7.22 (1H, d, *J* = 2.1 Hz, H‐6). ^13^C NMR (DMSO, 101 MHz) *δ* 159.61 (C‐7), 149.45 (C‐2′), 143.33 (C‐1), 138.71 (C‐5), 136.44 (C‐4^′^), 135.78 (C‐3), 133.55 (C‐10’), 130.09 (C‐8^′^), 129.67 (q, *J* = 32.1 Hz, C‐6^′^), 126.20 (q, *J* = 4.0 Hz, C‐5^′^), 125.10 (d, *J* = 3.9 Hz, C‐7^′^), 124.60 (C‐4), 123.90 (q, *J* = 272.3 Hz, CF_3_), 114.13 (C‐6), 110.48 (C‐2). HRMS (ESI+): *m/z* [M + Na]^+^ calculated for C_15_H_10_O_2_N_3_Cl_2_F_3_Na = 413.9994, found: 413.9998.

##### (4‐Amino‐2‐(Trifluoromethyl)Phenyl)(Pyrrolid‐1‐Yl)Methanone (32)


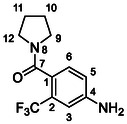


Compound **26** (200 mg, 0.98 mmol, 1.0 eq), pyrrolidine (208 mg, 2.93 mmol, 3.0 eq), Et_3_N (0.41 mL, 2.93 mmol, 3.0 eq), and HATU (482 mg, 1.27 mmol, 1.3 eq) were treated according to Method C to give **32** (220 mg, 87%) as a white solid. ^1^H NMR (DMSO, 401 MHz) *δ* 7.07 (1H, d, *J* = 7.4 Hz, H‐6), 6.87 (1H, d, *J* = 2.2 Hz, H‐3), 6.78 (1H, dd, *J* = 8.3, 2.3 Hz, H‐5), 5.74 (2H, s, NH_2_), 3.39 (2H, t, *J* = 6.9 Hz, H‐12), 3.06 (2H, t, *J* = 6.6 Hz, H‐9), 1.90—1.71 (4H, m, H‐10, H‐11). ^13^C NMR (DMSO, 101 MHz) *δ* 167.00 (C‐7), 149.36 (C‐4), 128.43 (C‐6), 125.95 (q, *J* = 30.5 Hz, C‐2), 124.01 (q, *J* = 273.2 Hz, CF_3_), 122.96 (q, *J* = 2.4 Hz, C‐1), 116.46 (C‐5), 110.22 (q, *J* = 4.9 Hz, C‐3), 48.16 (C‐9), 45.14 (C‐12), 25.45 (C‐10), 24.10 (C‐11). MS (ESI+): *m/z* [M + H]^+^ calculated for C_12_H_14_ON_2_F_3_ = 259.1, found: 259.2.

##### (4‐Amino‐2‐(Trifluoromethyl)Phenyl)(Morpholino)Methanone (33)


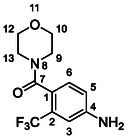


Compound **26** (200 mg, 0.98 mmol, 1.0 eq), morpholine (255 mg, 2.93 mmol, 3.0 eq), Et_3_N (0.41 mL, 2.93 mmol, 3.0 eq), and HATU (482 mg, 1.27 mmol, 1.3 eq) were treated according to Method C to give **33** (234 mg, 88%) as a white solid. ^1^H NMR (DMSO, 401 MHz) *δ* 7.05 (1H, d, *J* = 8.3 Hz, H‐6), 6.89 (1H, d, *J* = 2.3 Hz, H‐3), 6.79 (1H, dd, *J* = 8.3, 2.3 Hz, H‐5), 5.79 (2H, s, NH_2_), 3.70—3.36 (6H, m, H‐10, H‐12, H‐13), 3.23—3.01 (2H, m, H‐9). ^13^C NMR (DMSO, 101 MHz) *δ* 167.39 (C‐7), 149.61 (C‐4), 128.58 (C‐6), 126.52 (q, *J* = 30.6 Hz, C‐2), 123.98 (q, *J* = 273.8 Hz, CF_3_), 120.66 (q, *J* = 2.4 Hz, C‐1), 116.41 (C‐5), 110.30 (q, *J* = 4.8 Hz, C‐3), 65.94 (C‐12), 65.85 (C‐10), 47.25 (C‐9), 41.69 (C‐13). MS (ESI+): *m/z* [M + H]^+^ calculated for C_12_H_14_O_2_N_2_F_3_ = 275.1, found: 275.2.

##### 4‐(Pyrrolidin‐1‐Ylmethyl)‐3‐(Trifluoromethyl)Aniline (34)


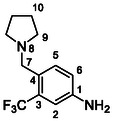


Compound **32** (130 mg, 0.50 mmol, 1.0 eq) was dissolved in dry THF (5.0 mL) and LiAlH_4_ (96 mg, 2.52 mmol, 5.0 eq) was added. The reaction mixture was heated to 50 °C for 3 h. 1 M aqueous solution of NaOH was added, and the mixture was extracted with DCM (3 × 10 mL). Combined organic fractions were evaporated, and the residue was dissolved in DMF, applied on flash chromatography column, and separated (RP ‐ C18aq, eluent water/methanol, gradient 0‐100%) to obtain **34** (70 mg, 57%) as a dark brown solid. ^1^H NMR (DMSO, 401 MHz) *δ* 7.30 (1H, d, *J* = 8.3 Hz, H‐5), 6.84 (1H, d, *J* = 2.4 Hz, H‐2), 6.74 (1H, dd, *J* = 8.3, 2.4 Hz, H‐6), 5.41 (1H, s, NH_2_), 3.53—3.50 (2H, m, H‐7), 2.43—2.37 (4H, m, H‐9), 1.70—1.65 (4H, m, H‐10). ^13^C NMR (DMSO, 101 MHz) *δ* 147.52 (q, *J* = 5.5 Hz, C‐1), 131.55 (C‐5), 127.20 (q, *J* = 29.0 Hz, C‐3), 124.79 (q, *J* = 274.4 Hz, CF_3_), 123.95 (C‐4), 116.88 (C‐6), 110.16 (q, *J* = 6.4 Hz, C‐2), 55.11 (C‐7), 53.53 (C‐9), 23.14 (C‐10). MS (ESI+): *m/z* [M + H]^+^ calculated for C_12_H_16_N_2_F_3_ = 245.1, found: 245.3.

##### 4‐(Morpholinomethyl)‐3‐(Trifluoromethyl)Aniline (35)


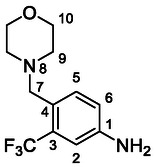


Compound **33** (130 mg, 0.47 mmol, 1.0 eq) was dissolved in dry THF (5.0 mL), and LiAlH_4_ (54 mg, 1.42 mmol, 3.0 eq) was added. The reaction mixture was heated to 50 °C for 1 h. The reaction mixture was diluted with 1 M aqueous solution of NaOH (10 mL) and extracted with DCM (3 × 10 mL). Combined organic fractions were evaporated, and the residue was dissolved in DMF, applied on flash chromatography column, and separated (RP ‐ C18aq, eluent water/methanol, gradient 0‐100%) to obtain **35** (58 mg, 47%) as a yellowish solid. ^1^H NMR (DMSO, 401 MHz) *δ* 7.30 (1H, d, *J* = 8.3 Hz, H‐5), 6.86 (1H, d, *J* = 2.4 Hz, H‐2), 6.75 (1H, dd, *J* = 8.4, 2.4 Hz, H‐6), 5.45 (2H, s, NH_2_), 3.55 (4H, t, *J* = 4.6 Hz, H‐10), 3.39 (2H, s, H‐7), 2.34—2.28 (4H, m, H‐9). ^13^C NMR (DMSO, 101 MHz) *δ* 147.91 (C‐1), 131.96 (C‐5), 127.96 (q, *J* = 28.9 Hz, C‐3), 124.75 (q, *J* = 274.1 Hz, CF_3_), 122.21 (q, *J* = 1.6 Hz, C‐4), 116.84 (C‐6), 110.37 (q, *J* = 5.9 Hz, C‐2), 66.27 (C‐10), 58.04 (C‐7), 53.26 (C‐9). MS (ESI+): *m/z* [M + H]^+^ calculated for C_12_H_16_ON_2_F_3_ = 261.1, found: 261.2.

##### 1‐(3,5‐Dichlorophenyl)‐3‐(4‐(Pyrrolidin‐1‐Ylmethyl)‐3‐(Trifluoromethyl)Phenyl)Urea Hydrochloride (38)


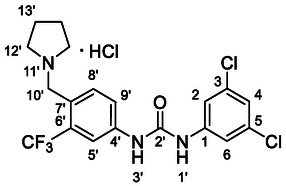


Compounds **12** (97 mg of the crude mixture, 0.16 mmol, 1.0 eq) and **34** (40 mg, 0.16 mmol, 1.0 eq) were treated according to Method B, followed by Method E, to give **38** (31 mg, 44%) as a yellow solid. ^1^H NMR (DMSO, 401 MHz) *δ* 10.63 (1H, s, H‐11’), 10.14—10.07 (2H, m, H‐1^′^, H‐3^′^), 8.05 (1H, d, *J* = 2.3 Hz, H‐5^′^), 8.01 (1H, d, *J* = 8.6 Hz, H‐8^′^), 7.70 (1H, dd, *J* = 8.5, 2.3 Hz, H‐9^′^), 7.53 (2H, d, *J* = 1.9 Hz, H‐2, H‐6), 7.19 (1H, t, *J* = 1.8 Hz, H‐4), 4.45 (2H, s, H‐10’), 3.51—3.40 (2H, m, H‐12’a), 3.17—3.04 (2H, m, H‐12’b), 2.08—1.86 (4H, m, H‐13’). ^13^C NMR (DMSO, 101 MHz) *δ* 152.34 (C‐2′), 141.92 (C‐1), 140.58 (C‐7^′^), 134.18 (C‐4^′^), 133.32 (C‐8^′^), 123.79 (q, *J* = 274.1 Hz, CF_3_), 121.52 (C‐9^′^), 121.21 (C‐4), 116.17 (C‐2, C‐6), 115.03 (q, *J* = 6.1 Hz, C‐5^′^), 53.44 (C‐12’), 53.02 (C‐10’), 22.55 (C‐13’). HRMS (ESI+): *m/z* [M + H]^+^ calculated for C_19_H_19_ON_3_Cl_2_F_3_ = 432.0852, found: 432.0850.

##### 1‐(3,5‐Dichlorophenyl)‐3‐(4‐(Morpholinomethyl)‐3‐(Trifluoromethyl)Phenyl)Urea Hydrochloride (39)


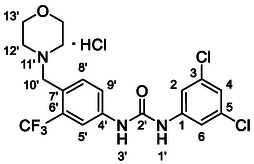


Compounds **12** (69 mg of the crude mixture), 0.12 mmol, 1.0 eq) and **35** (30 mg, 0.12 mmol, 1.0 eq) were treated according to Method B, followed by Method E, to give **39** (14 mg, 27%) as a yellowish solid. ^1^H NMR (DMSO, 401 MHz) *δ* 10.69 (1H, s, H‐11’), 10.09—9.94 (2H, m, H‐1^′^, H‐3^′^), 8.12—8.03 (2H, m, H‐5^′^, H‐9^′^), 7.76—7.69 (1H, m, H‐8^′^), 7.53 (2H, d, *J* = 1.9 Hz, H‐6), 7.19 (1H, t, *J* = 1.8 Hz, H‐4), 4.43 (2H, s, H‐10’), 3.98—3.77 (4H, m, H‐13’), 3.34—3.14 (4H, m, H‐12’). ^13^C NMR (DMSO, 101 MHz) *δ* 152.31 (C‐2′), 141.89 (C‐1), 134.42 (C‐8^′^), 134.18 (C‐3, C‐5), 121.49 (C‐9^′^), 121.26 (C‐4), 116.26 (C‐2, C‐6), 115.14 (q, *J* = 5.9 Hz, C‐5^′^), 63.08 (C‐13’), 55.53 (C‐10’), 51.47 (C‐12’). HRMS (ESI+): *m/z* [M + H]^+^ calculated for C_19_H_19_O_2_N_3_Cl_2_F_3_ = 448.0801, found: 448.0800.

##### 1‐(3,5‐Dichlorophenyl)‐3‐(4‐((4‐Methylpiperazin‐1‐Yl)Methyl)‐3‐(Trifluoromethyl)Phenyl)Urea Hydrochloride (40)


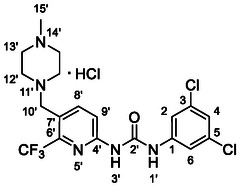


Compound **12** (313 mg of the crude mixture, 0.53 mmol, 1.0 eq) and 4‐((4‐methylpiperazin‐1‐yl)methyl)‐3‐(trifluoromethyl)aniline (145 mg, 0.53 mmol, 1.0 eq) were treated according to Method B, followed by Method E, to give **40** (100 mg, 41%) as a white solid. ^1^H NMR (DMSO, 401 MHz) *δ* 10.07 (2H, s, H‐1^′^, H‐3^′^), 8.01 (1H, d, *J* = 2.2 Hz, H‐5^′^), 7.99—7.93 (1H, m, H‐8^′^), 7.68 (1H, dd, *J* = 8.4, 2.3 Hz, H‐9^′^), 7.53 (2H, d, *J* = 1.9 Hz, H‐2, H‐6), 7.18 (1H, t, *J* = 1.9 Hz, H‐4), 4.16 (2H, s, H‐10’), 3.63—3.49 (2H, m, H‐13’a), 3.43—3.08 (6H, m, H‐12’, H‐13’b), 2.80 (3H, s, H‐15’). ^13^C NMR (DMSO, 101 MHz) *δ* 152.35 (C‐2′), 141.97 (C‐1), 134.18 (C‐3, C‐5), 133.13 (C‐8^′^), 123.89 (q, *J* = 273.3 Hz, CF_3_), 121.47 (C‐9^′^), 121.15 (C‐4), 116.13 (C‐2, C‐6), 115.04 (q, *J* = 6.2 Hz, C‐5^′^), 55.24 (C‐10’), 50.30 (C‐13’), 48.49 (C‐12’), 41.79 (C‐15’). HRMS (ESI+): *m/z* [M + H]^+^ calculated for C_20_H_22_ON_4_Cl_2_F_3_ = 461.1117, found: 461.1118.

##### 1‐(4‐(Aminomethyl)‐3‐(Trifluoromethyl)Phenyl)‐3‐(3,5‐Dichlorophenyl)Urea Hydrochloride (41)


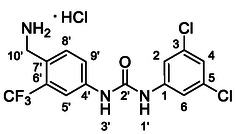


4‐Amino‐2‐trifluoromethylbenzyl amine (105 mg, 0.55 mmol, 1.0 eq) was dissolved in THF (5.0 mL), and Boc_2_O (241 mg, 1.10 mmol, 2.0 eq) with Et_3_N (0.23 mL, 1.66 mmol, 3.0 eq) were added. After stirring at RT for 1 h, the solvent was removed under reduced pressure, the solid residue was dissolved in DCM (5.0 mL), and **12** (322 mg of the crude mixture, 0.55 mmol, 1.0 eq) was added. The reaction mixture was heated to 60 °C for 12 h. The reaction mixture was evaporated, the solid residue was dissolved in TFA (2.0 mL) and stirred or 15 min at RT. TFA was removed under reduced pressure, and the crude product was applied on flash chromatography column and separated (RP ‐ C18aq, eluent water/methanol, gradient 0–100%), followed by Method E, to obtain **41** (76 mg, 36%) as a white solid. ^1^H NMR (DMSO, 401 MHz) *δ* 10.00 (1H, s, H‐1^′^), 9.99 (1H, s, H‐3^′^), 8.44 (3H, s, NH_2_), 8.02 (1H, d, *J* = 1.9 Hz, H‐5^′^), 7.73—7.65 (2H, m, H‐8^′^, H‐9^′^), 7.53 (2H, d, *J* = 1.8 Hz, H‐2, H‐6), 7.19 (1H, t, *J* = 1.9 Hz, H‐4), 4.10 (2H, s, H‐10’). ^13^C NMR (DMSO, 101 MHz) *δ* 152.35 (C‐2′), 141.95 (C‐1), 140.11 (C‐4^′^), 134.17 (C‐3, C‐5), 132.01 (C‐8^′^), 127.88 (q, *J* = 29.8 Hz, C‐6^′^), 124.67 (C‐7^′^), 123.88 (q, *J* = 274.0 Hz, CF_3_), 121.57 (C‐9^′^), 121.19 (C‐4), 116.19 (C‐2, C‐6), 115.05 (q, *J* = 6.0 Hz, C‐5^′^), 38.38 (C‐10’). HRMS (ESI+): *m/z* [M + H]^+^ calculated for C_15_H_13_ON_3_Cl_2_F_3_ = 378.0382, found: 378.0383.

##### (5‐Amino‐2‐(Trifluoromethyl)Phenyl)(Pyrrolid‐1‐Yl)Methanone (43)


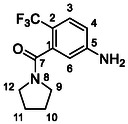


Compound **42** (50 mg, 0.24 mmol, 1.0 eq), pyrrolidine (35 mg, 0.49 mmol, 2.0 eq), Et_3_N (0.10 mL, 0.73 mmol, 3.0 eq), and HATU (121 mg, 0.32 mmol, 1.3 eq) were treated according to Method C to give **43** (60 mg, 95%) as a yellowish oil. ^1^H NMR (DMSO, 401 MHz) *δ* 7.36 (1H, s, H‐3), 6.67—6.61 (1H, m, H‐4), 6.44 (1H, d, *J* = 2.3 Hz, H‐6), 5.95 (2H, s, NH_2_), 3.40 (2H, t, *J* = 6.9 Hz, H‐12), 3.05 (2H, t, *J* = 6.6 Hz, H‐9), 1.90—1.72 (4H, m, H‐10, H‐11). ^13^C NMR (DMSO, 101 MHz) *δ* 166.69 (C‐7), 152.26 (C‐1), 138.03—137.18 (m, C‐5), 127.66 (q, *J* = 4.6 Hz, C‐3), 124.83 (q, *J* = 271.1 Hz, CF_3_), 112.80 (C‐4), 110.73 (C‐6), 110.65 (q, *J* = 31.6 Hz, C‐2), 47.99 (C‐9), 45.02 (C‐12), 25.38 (C‐10), 24.04 (C‐11). MS (ESI+): *m/z* [M + H]^+^ calculated for C_12_H_14_ON_2_F_3_ = 259.1, found: 259.2.

##### (5‐Amino‐2‐(Trifluoromethyl)Phenyl)(Morpholino)Methanone (44)


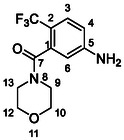


Compound **42** (50 mg, 0.24 mmol, 1.0 eq), morpholine (64 mg, 0.73 mmol, 3.0 eq), Et_3_N (0.10 mL, 0.73 mmol, 3.0 eq), and HATU (121 mg, 0.32 mmol, 1.3 eq) were treated according to Method C to give **44** (62 mg, 93%) as a yellowish oil. ^1^H NMR (DMSO, 401 MHz) *δ* 7.36 (1H, d, *J* = 8.6 Hz, H‐3), 6.65 (2H, dd, *J* = 8.6, 1.5 Hz, H‐4), 6.44 (1H, d, *J* = 2.2 Hz, H‐6), 6.00 (2H, s, NH_2_), 3.65—3.54 (4H, m, H‐12, H‐13), 3.52—3.43 (2H, m, H‐10), 3.20—3.02 (2H, m, H‐9). ^13^C NMR (DMSO, 101 MHz) *δ* 167.01 (C‐7), 152.22 (C‐5), 135.67 (q, *J* = 2.4 Hz, C‐1), 127.74 (q, *J* = 4.5 Hz, C‐3), 124.82 (q, *J* = 271.1 Hz, CF_3_), 113.01 (C‐4), 111.09 (q, *J* = 31.6 Hz, C‐2), 110.68 (C‐6), 65.95 (C‐10), 65.81 (C‐12), 47.00 (C‐9), 41.43 (C‐13). MS (ESI+): *m/z* [M + H]^+^ calculated for C_12_H_14_O_2_N_2_F_3_ = 275.1, found: 275.2.

##### (5‐Amino‐2‐(Trifluoromethyl)Phenyl)(4‐Methylpiperazin‐1‐Yl)Methanone (45)


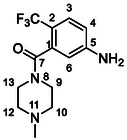


Compound **42** (100 mg, 0.49 mmol, 1.0 eq), *N*methylpiperazine (147 mg, 1.46 mmol, 3.0 eq), Et_3_N (0.20 mL, 1.46 mmol, 3.0 eq), and HATU (241 mg, 0.63 mmol, 1.3 eq) were treated according to Method C to give **45** (135 mg, 96%) as a colorless oil. ^1^H NMR (DMSO, 401 MHz) *δ* 7.35 (1H, d, *J* = 8.6 Hz, H‐3), 6.64 (1H, dd, *J* = 8.7, 1.3 Hz, H‐4), 6.41 (1H, d, *J* = 2.3 Hz, H‐6), 5.99 (2H, s, NH_2_), 3.68—3.46 (2H, m, H‐13), 3.18—3.00 (2H, m, H‐9), 2.37—2.18 (4H, m, H‐10, H‐12), 2.18 (3H, s, CH_3_). ^13^C NMR (DMSO, 101 MHz) *δ* 166.79 (C‐7), 152.17 (C‐5), 136.04 (q, *J* = 2.4 Hz, C‐1), 127.70 (q, *J* = 4.7 Hz, C‐3), 124.81 (q, *J* = 271.3 Hz, CF_3_), 112.91 (C‐4), 111.05 (q, *J* = 31.5 Hz, C‐2), 110.62 (C‐6), 54.22 (C‐10), 54.12 (C‐12), 46.45 (C‐9), 45.64 (CH_3_), 40.81 (C‐13). MS (ESI+): *m/z* [M + H]^+^ calculated for C_13_H_17_ON_3_F_3_ = 288.1, found: 288.2.

##### 1‐(3,5‐Dichlorophenyl)‐3‐(3‐(Pyrrolidin‐1‐Ylmethyl)‐4‐(Trifluoromethyl)Phenyl)Urea Hydrochloride (46)


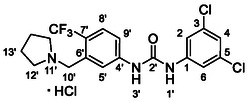


Synhydrid (0.44 mL of 3.5 M solution in toluene, 1.55 mmol, 8.0 eq) was added dropwise to a solution of **43** (50 mg, 0.19 mmol, 1.0 eq) in dry THF (5.0 mL), and the mixture was stirred at 65 °C for 3 days. The reaction mixture was cooled down to 0 °C, followed by the addition of a 1 M aqueous solution of NaOH, and the mixture was extracted with DCM (3 × 10 mL). Combined organic fractions were evaporated, and the residue was treated with compound **12** (110 mg of the crude mixture, 0.19 mmol, 1.0 eq) according to Method B, followed by Method E, to give **46** (15 mg, 16%) as a white solid. ^1^H NMR (DMSO, 401 MHz) *δ* 10.38 (1H, s, H‐1^′^), 10.33 (1H, s, H‐11’), 9.99 (1H, s, H‐3^′^), 8.01 (1H, d, *J* = 2.0 Hz, H‐5^′^), 7.77 (1H, d, *J* = 8.8 Hz, H‐8^′^), 7.72 (1H, dd, *J* = 8.7, 2.0 Hz, H‐9^′^), 7.55 (2H, d, *J* = 1.9 Hz, H‐2, H‐6), 7.20 (1H, t, *J* = 1.8 Hz, H‐4), 4.51 (2H, d, *J* = 5.5 Hz, H‐10’), 3.55—3.43 (2H, m, 12’a), 3.22—3.09 (2H, m, 12’b), 2.04 (2H, q, *J* = 7.1 Hz, 13’a), 1.92 (2H, pd, *J* = 7.5, 3.0 Hz, 13’b). ^13^C NMR (DMSO, 101 MHz) *δ* 152.23 (C‐2′), 143.36 (C‐1), 141.92 (C‐4^′^), 134.21 (C‐3, C‐5), 130.49 (C‐6^′^), 127.74 (d, *J* = 5.5 Hz, C‐8^′^), 124.18 (d, *J* = 272.9 Hz, CF_3_), 121.23 (C‐5^′^), 121.00 (C‐4), 120.98 (d, *J* = 29.6 Hz, C‐7^′^), 118.43 (C‐9^′^), 116.09 (C‐2, C‐6), 53.83 (C‐12’), 53.38 (C‐10’), 22.50 (C‐13’). HRMS (ESI+): *m/z* [M + H]^+^ calculated for C_19_H_19_ON_3_Cl_2_F_3_ = 432.0852, found: 432.0850.

##### 1‐(3,5‐Dichlorophenyl)‐3‐(3‐(Morpholinomethyl)‐4‐(Trifluoromethyl)Phenyl)Urea Hydrochloride (47)


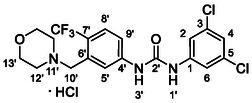


Synhydrid (0.20 mL of 3.5 M solution in toluene, 0.73 mmol, 8.0 eq) was added dropwise to a solution of **44** (25 mg, 0.09 mmol, 1.0 eq) in dry THF (5.0 mL), and the mixture was stirred at 65 °C for 3 days. The reaction mixture was cooled down to 0 °C, followed by the addition of a 1 M aqueous solution of NaOH, and the mixture was extracted with DCM (3 × 10 mL). Combined organic fractions were evaporated and the residue was treated with compound **12** (52 mg of the crude mixture, 0.09 mmol, 1.0 eq) according to Method B, followed by Method E, to give **47** (17 mg, 44%) as a white solid. ^1^H NMR (DMSO, 401 MHz) *δ* 9.37 (1H, s, H‐1^′^), 9.13 (1H, s, H‐3^′^), 7.77 (1H, d, *J* = 2.1 Hz, H‐5^′^), 7.66—7.58 (2H, m, H‐8^′^, H‐9^′^), 7.54 (2H, d, *J* = 1.9 Hz, H‐2, H‐6), 7.19 (1H, t, *J* = 1.9 Hz, H‐4), 3.61 (4H, t, *J* = 4.6 Hz, H‐13’), 3.57 (2H, s, H‐10’), 2.40 (4H, dd, *J* = 5.7, 3.3 Hz, H‐12’). ^13^C NMR (DMSO, 101 MHz) *δ* 152.05 (C‐2′), 142.90 (C‐4^′^), 141.94 (C‐1), 138.01 (C‐6^′^), 134.11 (C‐3, C‐5), 126.91 (q, *J* = 5.9 Hz, C‐8^′^), 124.69 (q, *J* = 272.8 Hz, CF_3_), 121.27 (C‐4), 120.58 (q, *J* = 29.9 Hz, C‐7^′^), 119.41 (C‐5^′^), 116.57 (C‐2, C‐6), 116.15 (C‐9^′^), 66.26 (C‐13’), 58.05 (C‐10’), 53.32 (C‐12’). HRMS (ESI+): *m/z* [M + H]^+^ calculated for C_19_H_19_O_2_N_3_Cl_2_F_3_ = 448.0801, found: 448.0803.

##### 1‐(3,5‐Dichlorophenyl)‐3‐(3‐((4‐Methylpiperazin‐1‐Yl)Methyl)‐4‐(Trifluoromethyl)Phenyl)Urea Hydrochloride (48)


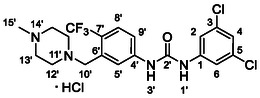


Compound **45** (130 mg, 0.45 mmol, 1.0 eq) was dissolved in dry THF (5.0 mL) and LiAlH_4_ (69 mg, 1.81 mmol, 4.0 eq) was added. The reaction mixture was heated to 50 °C for 4 h. 1 M aqueous solution of NaOH was added, and the mixture was extracted with DCM (3 × 10 mL). Combined organic fractions were evaporated and the residue was treated with compound **12** (260 mg of the crude mixture, 0.45 mmol, 1.0 eq) according to Method B, followed by Method E, to give **48** (8 mg, 4%) as a white solid. ^1^H NMR (DMSO, 401 MHz) *δ* 10.09 (1H, s, H‐1^′^), 9.97 (1H, s, H‐3^′^), 8.25 (1H, s, NH^+^), 7.80 (1H, d, *J* = 2.1 Hz, H‐5^′^), 7.65—7.59 (2H, m, H‐8^′^, H‐9^′^), 7.58 (2H, d, *J* = 1.9 Hz, H‐2, H‐6), 7.16 (1H, t, *J* = 1.9 Hz, H‐4), 3.56 (2H, s, H‐10’), 2.46 (8H, s, 12’, H‐13’), 2.27 (3H, s, H‐15’). ^13^C NMR (DMSO, 101 MHz) *δ* 152.35 (C‐2′), 143.28 (C‐1), 142.38 (C‐4^′^), 138.18 (C‐6^′^), 134.07 (C‐3, C‐5), 126.81 (d, *J* = 5.8 Hz, C‐8^′^), 124.75 (d, *J* = 272.6 Hz, CF_3_), 120.99 (C‐4), 120.34 (d, *J* = 30.4 Hz, C‐7^′^), 119.44 (C‐5^′^), 116.44 (C‐2, C‐6), 116.16 (C‐9^′^), 57.62 (C‐10’), 54.40 (C‐13’), 52.18 (C‐12’), 45.12 (C‐15’). HRMS (ESI+): *m/z* [M + H]^+^ calculated for C_20_H_22_ON_4_Cl_2_F_3_ = 461.1117, found: 461.1115.

##### 4‐Amino‐2,6‐Dichlorobenzaldehyde (50)


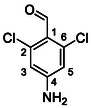


n‐Butyllithium (2.7 mL of 2.5 M solution in toluene, 6.8 mmol, 1.1 eq) was added dropwise to the solution of 3,5‐dichloroaniline (1.0 g, 6.2 mmol, 1.0 eq) in dry THF (20 mL) at −78 °C. After 20 min, TMSCl (0.86 mL, 6.8 mmol, 1.1 eq) was added dropwise, and the mixture was allowed to warm to −20 °C for 20 min. The mixture was cooled to −78 °C, n‐butyllithium (2.7 mL of 2.5 M solution in toluene, 6.8 mmol, 1.1 eq) was added dropwise, and after 20 min, TMSCl (0.86 mL, 6.8 mmol, 1.1 eq) was added dropwise, and the mixture was allowed to warm to −20 °C for 20 min. Then, the solution was cooled to −78 °C again, n‐butyllithium (2.7 mL of 2.5 M solution in toluene, 6.8 mmol, 1.1 eq) was added dropwise, and, after 20 min, dry DMF (0.52 mL, 6.8 mmol, 1.1 eq) was added dropwise. Aqueous 1 M HCl was added to the reaction mixture till acidic pH, water (20 mL) was added, and the mixture was extracted with DCM (3 × 10 mL). Combined organic fractions were evaporated, and the residue was dissolved in DMF, applied on flash chromatography column, and separated (RP ‐ C18aq, eluent water/methanol, gradient 0–100%) to obtain **50** (0.8 g, 68%) as a yellow solid. ^1^H NMR (401 MHz, DMSO) *δ* 10.11 (s, 1H, C**H**O), 6.80 (s, 2H, NH_2_), 6.63 (s, 2H, H‐3, H‐5). ^13^C NMR (DMSO, 101 MHz) *δ* 185.77 (**C**HO), 154.29 (C‐4), 138.44 (C‐2, C‐6), 115.85 (C‐1), 113.46 (C‐3, C‐5). MS (ESI+): *m/z* [M + H]^+^ calculated for C_7_H_6_ONCl_2_ = 190.0, found: 190.1.

##### 3,5‐Dichloro‐4‐((4‐Methylpiperazin‐1‐Yl)Methyl)Aniline (52) and 4‐Amino‐2,6‐Dichlorophenyl)Methanol (53)


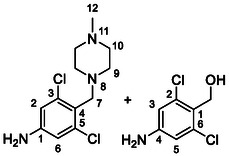


*N*‐Methylpiperazine (158 mg, 1.58 mmol, 2.0 eq) and NaBH_3_CN (149 mg, 2.37 mmol, 3.0 eq) were added to the solution of **50** (150 mg, 0.79 mmol, 1.0 eq) in methanol (10 mL). The reaction mixture was stirred at RT for 12 h. The solvent was removed under reduced pressure, and the residue was dissolved in DMF, applied on flash chromatography column, and separated (RP ‐ C18aq, eluent water/methanol, gradient 0‐100%) to obtain **52** (90 mg, 42%) as a yellowish solid and **53** (72 mg, 48%) as a yellowish solid. Compound **52**: ^1^H NMR (DMSO, 401 MHz) *δ* 6.58 (2H, s, H‐2, H‐6), 5.61 (2H, s, NH_2_), 3.46 (2H, s, H‐7), 2.40 (4H, s, H‐9), 2.30—2.19 (4H, m, H‐10), 2.11 (3H, s, H‐12). ^13^C NMR (DMSO, 101 MHz) *δ* 149.50 (C‐1), 136.16 (C‐3, C‐5), 119.28 (C‐4), 112.98 (C‐2, C‐6), 55.45 (C‐7), 54.76 (C‐10), 52.34 (C‐9), 45.75 (C‐12). MS (ESI+): *m/z* [M + H]^+^ calculated for C_12_H_18_N_3_Cl_2_ = 274.1, found: 274.2. Compound **53**: ^1^H NMR (DMSO, 401 MHz) *δ* 6.58 (2H, s, H‐3, H‐5), 5.65 (2H, s, NH_2_), 4.80 (1H, s, OH), 4.50 (2H, s, CH_2_). ^13^C NMR (DMSO, 101 MHz) *δ* 149.85 (C‐4), 135.62 (C‐2, C‐6), 122.47 (C‐1), 112.76 (C‐3, C‐5), 57.71 (CH_2_). MS (ESI+): *m/z* [M + H]^+^ calculated for C_7_H_8_ONCl_2_ = 192.0, found: 192.2.

##### 1‐(3,5‐Dichloro‐4‐((4‐Methylpiperazin‐1‐Yl)Methyl)Phenyl)‐3‐(3‐(Trifluoromethyl)Phenyl)Urea Hydrochloride (54)


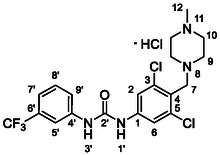


Compounds **6** (107 mg of the crude mixture, 0.16 mmol, 1.0 eq) and **52** (44 mg, 0.16 mmol, 1.0 eq) were treated according to Method B, followed by Method E, to give **54** (38 mg, 70%). ^1^H NMR (DMSO, 401 MHz) *δ* 11.16 (1H, s, NH^+^), 10.26 (1H, s, H‐1^′^), 10.03 (1H, s, H‐3^′^), 7.97 (1H, dd, *J* = 2.0, 2.0 Hz, H‐5^′^), 7.65 (2H, s, H‐2, H‐6), 7.59 (1H, ddd, *J* = 8.5, 1.5, 1.5 Hz, H‐9^′^), 7.53 (1H, dd, *J* = 7.9, 7.9 Hz, H‐8^′^), 7.36—7.31 (1H, m, H‐7^′^), 4.15 (2H, s, H‐7), 3.55—3.43 (2H, m, H‐9a), 3.23 (6H, s, H‐9b, H‐10), 2.78 (3H, s, H‐12). ^13^C NMR (DMSO, 101 MHz) *δ* 152.37 (C‐2′), 140.09 (C‐1), 136.69 (C‐4^′^), 130.07 (C‐8^′^), 129.60 (q, *J* = 31.2 Hz, C‐6^′^), 124.16 (q, *J* = 272.2 Hz, CF_3_), 121.80 (C‐9^′^), 118.54 (q, *J* = 4.0 Hz, C‐7^′^), 117.25 (C‐2, C‐6), 114.02 (q, *J* = 4.2 Hz, C‐5^′^), 54.28 (C‐7), 50.66 (C‐9), 48.66 (C‐10), 41.58 (C‐12). HRMS (ESI+): *m/z* [M + H]^+^ calculated for C_20_H_22_ON_4_Cl_2_F_3_ = 461.1117, found: 461.1117.

##### 4‐Amino‐*N*‐(2‐Methoxyethyl)‐2‐(Trifluoromethyl)Benzamide (56)


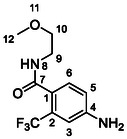


Compound **26** (3.00 g, 14.6 mmol, 1.0 eq), 2‐methoxyethylamine (1.43 g, 19.0 mmol, 1.3 eq), Et_3_N (4.1 mL, 29.3 mmol, 2.0 eq), and HATU (6.12 g, 16.09 mmol, 1.1 eq) were treated according to Method C to give **56** (3.10 g, 81%) as a white solid. ^1^H NMR (DMSO, 401 MHz) *δ* 8.15 (1H, t, *J* = 5.6 Hz, H‐8), 7.16 (1H, d, *J* = 8.3 Hz, H‐6), 6.87 (1H, d, *J* = 2.3 Hz, H‐3), 6.73 (1H, dd, *J* = 8.5, 2.5 Hz, H‐5), 5.79 (2H, s, NH_2_), 3.41—3.37 (2H, m, H‐10), 3.33—3.28 (2H, m, H‐9), 3.25 (3H, s, H‐12). ^13^C NMR (DMSO, 101 MHz) *δ* 167.74 (C‐7), 149.95 (C‐4), 129.96 (C‐6), 127.33 (q, *J* = 30.7 Hz, C‐2), 123.98 (q, *J* = 273.6 Hz, CF_3_), 123.00 (q, *J* = 2.5 Hz, C‐1), 115.44 (C‐5), 110.55 (q, *J* = 5.4 Hz, C‐3), 70.34 (C‐10), 57.93 (C‐12), 38.78 (C‐9). MS (ESI+): *m/z* [M + H]^+^ calculated for C_11_H_14_O_2_N_2_F_3_ = 263.1, found: 263.3.

##### 4‐(((2‐Methoxyethyl)Amino)Methyl)‐3‐(Trifluoromethyl)Aniline (57)


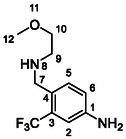


Compound **56** (3.1 g, 11.8 mmol, 1.0 eq) and synhydride (40.5 mL of 3.5 M solution in toluene, 141.9 mmol, 12.0 eq) were treated according to Method D to give **57** (1.27 g, 43%) as an orange oil. ^1^H NMR (DMSO, 401 MHz) *δ* 7.29 (1H, d, *J* = 8.3 Hz, H‐5), 6.85 (1H, d, *J* = 2.4 Hz, H‐2), 6.75 (1H, dd, *J* = 8.3, 2.2 Hz, H‐6), 5.40 (2H, s, NH_2_), 3.65 (2H, s, H‐7), 3.37 (2H, t, *J* = 5.6 Hz, H‐10), 3.21 (3H, s, H‐12), 2.62 (2H, t, *J* = 5.6 Hz, H‐9). ^13^C NMR (DMSO, 101 MHz) *δ* 147.61 (C‐1), 131.37 (C‐5), 127.25 (q, *J* = 28.9 Hz, C‐3), 125.05 (q, *J* = 1.7 Hz, C‐4), 124.88 (q, *J* = 274.0 Hz, CF_3_), 116.91 (C‐6), 110.26 (q, *J* = 6.0 Hz, C‐2), 71.67 (C‐10), 57.96 (C‐12), 48.64 (q, *J* = 2.0 Hz, C‐7), 47.94 (C‐9). MS (ESI+): *m/z* [M + H]^+^ calculated for C_11_H_16_ON_2_F_3_ = 249.1, found: 249.3.

##### 1‐(3,5‐Dichlorophenyl)‐3‐(4‐(((2‐Methoxyethyl)Amino)Methyl)‐3‐(Trifluoromethyl)Phenyl)Urea Hydrochloride (58)


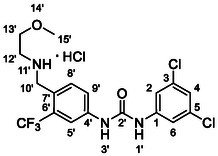


Compound **57** (40 mg, 0.16 mmol, 1.0 eq) was dissolved in THF (5.0 mL), and Boc_2_O (46 mg, 0.21 mmol, 1.3 eq) with Et_3_N (0.07 mL, 0.48 mmol, 3.0 eq) were added. The mixture was stirred at RT for 1 h. The solvent was removed under reduced pressure, the residue was dissolved in DCM (5.0 mL), and **12** (190 mg of the crude mixture, 0.32 mmol, 2.0 eq) was added. The reaction mixture was heated to 60 °C for 12 h, the solvent was evaporated, the residue dissolved in TFA (2.0 mL), and the mixture stirred at RT for 15 min. TFA was removed under reduced pressure, the residue was applied on flash chromatography column and separated (RP ‐ C18aq, eluent water/methanol, gradient 0‐100%), followed by Method E, to obtain **58** (65 mg, 85%) as a yellow solid. ^1^H NMR (DMSO, 401 MHz) *δ* 10.06 (2H, s, H‐1^′^, H‐3^′^), 9.26 (2H, s, H‐11’), 8.03 (1H, d, *J* = 2.3 Hz, H‐5^′^), 7.80 (1H, d, *J* = 8.5 Hz, H‐8^′^), 7.70 (1H, dd, *J* = 8.6, 2.3 Hz, H‐9^′^), 7.53 (2H, d, *J* = 1.9 Hz, H‐2, H‐6), 7.18 (1H, t, *J* = 1.8 Hz, H‐4), 4.28—4.22 (2H, m, H‐10’), 3.64 (2H, t, *J* = 5.1 Hz, H‐13’), 3.31 (3H, s, H‐15’), 3.23—3.15 (2H, m, H‐12’). ^13^C NMR (DMSO, 101 MHz) *δ* 152.35 (C‐2′), 141.95 (C‐1), 140.46 (C‐4^′^), 134.18 (C‐3, C‐5), 132.96 (C‐9^′^), 128.50 (q, *J* = 29.7 Hz, C‐6^′^), 123.82 (q, *J* = 274.4 Hz, CF_3_), 122.79 (C‐7^′^), 121.42 (C‐8^′^), 121.20 (C‐4), 116.18 (C‐2, C‐6), 115.03 (q, *J* = 6.1 Hz, C‐5^′^), 67.05 (C‐13’), 58.19 (C‐15’), 46.39 (C‐12’), 46.23 (C‐10’). HRMS (ESI+): *m/z* [M + H]^+^ calculated for C_18_H_19_O_2_N_3_Cl_2_F_3_ = 436.0801, found: 436.0798.

##### 1‐(3,5‐Dichlorophenyl)‐3‐(4‐(((2‐Hydroxyethyl)Amino)Methyl)‐3‐(Trifluoromethyl)Phenyl)Urea Hydrochloride (59)


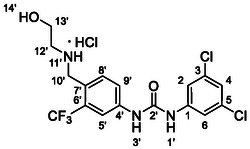


Compound **58** (20 mg, 0.04 mmol, 1.0 eq) was dissolved in DCM (50 mL), BBr_3_ (0.09 mL of 1 M solution in DCM, 0.09 mmol, 2.0 eq) was added dropwise, and the mixture was stirred for 1 h at RT. Water (0.05 mL) was added, the solvents were evaporated, and the residue was dissolved in DMSO, applied on flash chromatography column, and separated (RP ‐ C18aq, eluent water/methanol, gradient 0‐100%), followed by Method E, to obtain **59** (8 mg, 86%) as yellow solid. ^1^H NMR (DMSO, 401 MHz) *δ* 10.10—10.05 (2H, m, H‐1^′^, H‐3^′^), 9.21 (2H, s, H‐11’), 8.03 (1H, d, *J* = 2.3 Hz, H‐5^′^), 7.82 (1H, d, *J* = 8.5 Hz, H‐8^′^), 7.70 (1H, dd, *J* = 8.6, 2.3 Hz, H‐9^′^), 7.53 (2H, d, *J* = 1.9 Hz, H‐2, H‐6), 7.19 (1H, t, *J* = 1.9 Hz, H‐4), 5.34—5.20 (1H, m, H‐14’), 4.25 (2H, s, H‐10’), 3.73—3.69 (2H, m, H‐13’), 3.09—3.02 (2H, m, H‐12’). ^13^C NMR (DMSO, 101 MHz) *δ* 152.35 (C‐2′), 141.94 (C‐1), 140.42 (C‐4^′^), 134.18 (C‐3, C‐5), 132.92 (C‐8^′^), 128.48 (q, *J* = 29.7 Hz, C‐6^′^), 123.83 (q, *J* = 274.4 Hz, CF_3_), 122.85 (C‐7^′^), 121.41 (C‐9^′^), 121.19 (C‐4), 116.16 (C‐2, C‐6), 115.01 (q, *J* = 5.9 Hz, C‐5^′^), 56.38 (C‐13’), 49.21 (C‐12’), 46.17 (C‐10’). HRMS (ESI+): *m/z* [M + H]^+^ calculated for C_17_H_17_O_2_N_3_Cl_2_F_3_ = 422.0644, found: 422.0641.

##### Methyl (4‐Amino‐2‐(Trifluoromethyl)Benzyl)Glycinate (60)


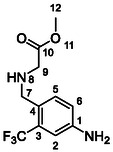


4‐Amino‐2‐trifluoromethylbenzyl amine (300 mg, 1.58 mmol, 1.0 eq) and Et_3_N (0.73 mL, 3.16 mmol, 2.0 eq) were dissolved in THF (10 mL). A solution of methyl bromoacetate (0.15 mL, 1.58 mmol, 1.0 eq) in THF (2.0 mL) was added to the first solution over 30 min at RT. The mixture was stirred for 4 h at RT, the solvent was removed under reduced pressure, and the residue was applied on a flash chromatography column and separated (RP ‐ C18aq, eluent water/methanol, gradient 0‐100%) to obtain **60** (348 mg, 84%) as a yellow oil. ^1^H NMR (DMSO, 401 MHz) *δ* 7.30 (1H, d, *J* = 8.4 Hz, H‐5), 6.85 (1H, d, *J* = 2.4 Hz, H‐2), 6.75 (1H, dd, *J* = 8.3, 2.4 Hz, H‐6), 5.43 (2H, s, NH_2_), 3.67 (2H, d, *J* = 1.7 Hz, H‐7), 3.61 (3H, s, H‐12), 3.30 (2H, s, H‐9). ^13^C NMR (DMSO, 101 MHz) *δ* 172.53 (C‐10), 147.73 (C‐1), 131.40 (C‐5), 127.37 (q, *J* = 28.9 Hz, C‐3), 124.77 (q, *J* = 274.0 Hz, CF_3_), 124.35 (q, *J* = 1.9 Hz, C‐4), 116.91 (C‐6), 110.19 (q, *J* = 6.0 Hz, C‐2), 51.23 (C‐12), 49.28 (C‐9), 47.65 (q, *J* = 2.0 Hz, C‐7). MS (ESI+): *m/z* [M + H]^+^ calculated for C_11_H_14_O_2_N_2_F_3_ = 263.1, found: 263.3.

##### Methyl (4‐(3‐(3,5‐Dichlorophenyl)Ureido)‐2‐(Trifluoromethyl)Benzyl)Glycinate Hydrochloride (61)


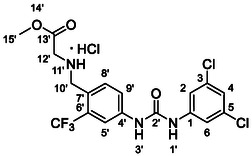


Compound **60** (70 mg, 0.27 mmol, 1.0 eq) was dissolved in THF (5.0 mL) and Boc_2_O (76 mg, 0.35 mmol, 1.3 eq) with Et_3_N (0.11 mL, 0.80 mmol, 3.0 eq) were added. The mixture was stirred at 60 °C for 2 h, the solvent was removed under reduced pressure, the residue was dissolved in DCM (5.0 mL), and **12** (313 mg of the crude mixture, 0.53 mmol, 2.0 eq) was added. The reaction mixture was heated to 60 °C for 12 h, the solvent was evaporated, and the residue was dissolved in TFA (2.0 mL) and stirred for 15 min at RT. TFA was removed under reduced pressure, and the residue was applied on flash chromatography column and separated (RP ‐ C18aq, eluent water/methanol, gradient 0‐100%), followed by Method E, to obtain **61** (105 mg, 87%) as a yellowish oil. ^1^H NMR (DMSO, 401 MHz) *δ* 10.00 (1H, s, H‐1^′^), 9.98 (1H, s, H‐3^′^), 9.69 (2H, s, H‐11’), 8.03 (1H, d, *J* = 2.3 Hz, H‐5^′^), 7.79 (1H, d, *J* = 8.6 Hz, H‐8^′^), 7.71 (1H, dd, *J* = 8.5, 2.3 Hz, H‐9^′^), 7.53 (2H, d, *J* = 1.9 Hz, H‐2, H‐6), 7.19 (1H, t, *J* = 1.9 Hz, H‐4), 4.30 (2H, s, H‐10’), 4.08 (2H, s, H‐12’), 3.77 (3H, s, H‐15’). ^13^C NMR (DMSO, 101 MHz) *δ* 166.99 (C‐13’), 152.33 (C‐2′), 141.92 (C‐1), 140.51 (C‐4^′^), 134.18 (C‐3, C‐5), 132.96 (C‐8^′^), 128.49 (q, *J* = 29.8 Hz, C‐6^′^), 123.75 (q, *J* = 274.5 Hz, CF_3_), 122.46 (C‐7^′^), 121.50 (C‐9^′^), 121.24 (C‐4), 116.24 (C‐2, C‐6), 115.11 (q, *J* = 6.2 Hz, C‐5^′^), 52.70 (C‐15’), 46.74 (C‐12’), 46.12 (q, *J* = 2.6 Hz, C‐10’). HRMS (ESI+): *m/z* [M + H]^+^ calculated for C_18_H_17_O_3_N_3_Cl_2_F_3_ = 450.0594, found: 450.0590.

##### Lithium (4‐(3‐(3,5‐Dichlorophenyl)Ureido)‐2‐(Trifluoromethyl)Benzyl)Glycinate (62)


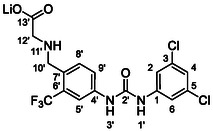


Compound **61** (35 mg, 0.08 mmol, 1.0 eq) was dissolved in a mixture of water (2.0 mL) and dioxane (2.0 mL), lithium hydroxide monohydrate (10 mg, 0.23 mmol, 3.0 eq) was added at RT, and the mixture was mixed at RT for 30 min. The reaction mixture was applied on a flash chromatography column and separated (RP ‐ C18aq, eluent water/acetonitrile, gradient 0‐100%) to obtain **62** (23 mg, 67%) as a yellow solid. ^1^H NMR (DMSO, 401 MHz) *δ* 10.49 (1H, s, H‐1^′^), 10.41 (1H, s, H‐3^′^), 8.00 (1H, d, *J* = 2.3 Hz, H‐5^′^), 7.89 (1H, d, *J* = 8.5 Hz, H‐8^′^), 7.68 (1H, dd, *J* = 8.5, 2.3 Hz, H‐9^′^), 7.52 (2H, d, *J* = 1.9 Hz, H‐2, H‐6), 7.16 (1H, t, *J* = 1.9 Hz, H‐4), 4.29 (2H, s, H‐10’), 3.90 (2H, s, H‐12’). ^13^C NMR (DMSO, 101 MHz) *δ* 167.73 (C‐13’), 152.45 (C‐2′), 142.04 (C‐1), 140.44 (C‐4^′^), 134.24 (C‐3, C‐5), 132.89 (C‐8^′^), 128.49 (q, *J* = 29.7 Hz, C‐6^′^), 123.79 (q, *J* = 274.0 Hz, CF_3_), 122.56 (C‐7^′^), 121.12 (C‐9^′^), 121.08 (C‐4), 115.91 (C‐2, C‐6), 114.72 (q, *J* = 5.9 Hz, C‐5^′^), 46.81 (C‐12’), 45.83 (C‐10’). HRMS (ESI‐): *m/z* [M−H]^−^ calculated for C_17_H_13_O_3_N_3_Cl_2_F_3_ = 434.0292, found: 434.0295.

##### 2‐((4‐Amino‐2‐(Trifluoromethyl)Benzyl)Amino)Ethan‐1‐Ol (63)


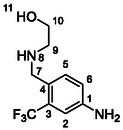


Compound **57** (400 mg, 1.61 mmol, 1.0 eq) was dissolved in DCM (50 mL), BBr_3_ (3.2 mL of 1 M solution in DCM, 3.22 mmol, 2.0 eq) was added dropwise, and the mixture stirred for 1 h. Water (0.25 mL) was added, solvents were evaporated, and the residue was dissolved in DMSO, applied on flash chromatography column, and separated (RP ‐ C18aq, eluent water/methanol, gradient 0‐100%) to obtain **63** (326 mg, 86%) as an orange solid. ^1^H NMR (DMSO, 401 MHz) *δ* 8.82—8.72 (2H, m, NH_2_), 7.45 (1H, d, *J* = 8.4 Hz, H‐5), 6.94 (1H, d, *J* = 2.4 Hz, H‐2), 6.83 (1H, dd, *J* = 8.4, 2.4 Hz, H‐6), 4.11 (2H, t, *J* = 5.2 Hz, H‐7), 3.68 (2H, t, *J* = 5.4 Hz, H‐10), 2.99 (2H, t, *J* = 5.5 Hz, H‐9). ^13^C NMR (DMSO, 101 MHz) *δ* 149.79 (C‐1), 133.54 (C‐5), 128.84 (q, *J* = 29.0 Hz, C‐3), 124.22 (q, *J* = 274.1 Hz, CF_3_), 116.56 (C‐6), 114.76 (q, *J* = 1.8 Hz, C‐4), 110.43 (q, *J* = 5.8 Hz, C‐2), 56.28 (C‐10), 48.73 (C‐9), 46.46 (q, *J* = 2.6 Hz, C‐7). MS (ESI+): *m/z* [M + H]^+^ calculated for C_10_H_14_ON_2_F_3_ = 235.1, found: 235.3.

##### 1‐(3,5‐Dichlorophenyl)‐3‐(4‐((2‐Oxomorpholino)Methyl)‐3‐(Trifluoromethyl)Phenyl)Urea (64)


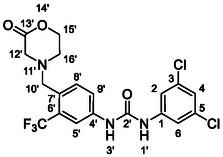


Compound **63** (80 mg, 0.34 mmol, 1.0 eq) and Et_3_N (0.14 mL, 1.03 mmol, 3.0 eq) were dissolved in dry acetonitrile (5.0 mL), methyl bromoacetate (63 mg, 0.41 mmol, 1.2 eq) was added dropwise at RT, and the mixture was stirred for 45 min. PTSA (260 mg, 1.37 mmol, 4.0 eq) was added, and after 2 h at RT, a complete conversion to lactone was observed (UPLC‐MS analysis). Afterwards, Et_3_N (0.14 mL, 1.03 mmol, 3.0 eq) and then **12** (200 mg of the crude mixture, 0.34 mmol, 1.0 eq) were added. The reaction mixture was heated to 60 °C for 12 h. The solvent was removed under reduced pressure, and the residue was dissolved in DMF, applied on flash chromatography column, and separated (RP ‐ C18aq, eluent water/acetonitrile, gradient 0‐100%) to obtain **64** (60 mg, 38%) as a white solid. ^1^H NMR (DMSO, 401 MHz) *δ* 9.34 (1H, s, H‐3^′^), 9.27 (1H, s, H‐1^′^), 7.97 (1H, d, *J* = 2.2 Hz, H‐5^′^), 7.68—7.60 (2H, m, H‐8^′^, H‐9^′^), 7.54 (2H, d, *J* = 1.9 Hz, H‐2, H‐6), 7.18 (1H, t, *J* = 1.8 Hz, H‐4), 4.36—4.29 (2H, m, H‐15’), 3.67 (2H, s, H‐10’), 3.34 (2H, s, H‐12’), 2.68 (2H, t, *J* = 5.1 Hz, H‐16’). ^13^C NMR (DMSO, 101 MHz) *δ* 167.06 (C‐13’), 152.28 (C‐2′), 142.01 (q, *J* = 2.2 Hz, C‐1), 138.81 (C‐4^′^), 134.10 (C‐3, C‐5), 131.65 (C‐8^′^), 128.91 (C‐7^′^), 127.69 (q, *J* = 29.7 Hz, C‐6^′^), 124.24 (q, *J* = 274.1 Hz, CF_3_), 122.03 (C‐9^′^), 121.18 (C‐4), 116.52 (C‐2, C‐6), 115.49 (q, *J* = 6.2 Hz, C‐5^′^), 68.60 (C‐15’), 55.83 (C‐10’), 55.14 (C‐12’), 47.85 (C‐16’). HRMS (ESI‐): *m/z* [M−H]^−^ calculated for C_19_H_15_O_3_N_3_Cl_2_F_3_ = 460.0448, found: 460.0451.

##### (4‐Amino‐2‐(Trifluoromethyl)Phenyl)Methanol (65)


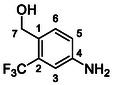


Compound **26** (0.4 g, 2.0 mmol, 1.0 eq) and synhydride (5.6 mL of 3.5 M solution in toluene, 19.5 mmol, 10 eq) were treated according to Method D to give **65** (224 mg, 66%) as a yellowish solid. ^1^H NMR (DMSO, 401 MHz) *δ* 7.32 (1H, d, *J* = 8.3 Hz, H‐6), 6.84 (1H, d, *J* = 2.4 Hz, H‐3), 6.77 (1H, dd, *J* = 8.3, 2.4 Hz, H‐5), 5.42 (2H, s, NH_2_), 5.04 (1H, t, *J* = 5.5 Hz, OH), 4.51—4.41 (2H, m, H‐7). ^13^C NMR (DMSO, 101 MHz) *δ* 147.78 (C‐4), 130.21 (C‐6), 126.58 (q, *J* = 1.7 Hz, C‐1), 126.26 (q, *J* = 28.0 Hz, C‐2), 124.76 (q, *J* = 272.5 Hz, CF_3_), 116.75 (C‐5), 110.14 (q, *J* = 5.8 Hz, C‐3), 59.05 (d, *J* = 2.7 Hz, C‐7). MS (ESI+): *m/z* [M + H]^+^ calculated for C_8_H_9_ONF_3_ = 192.1, found: 192.2.

##### Ethyl 1‐(4‐(3‐(3,5‐Dichlorophenyl)Ureido)‐2‐(Trifluoromethyl)Benzyl)Piperidine‐4‐Carboxylate Hydrochloride (67)


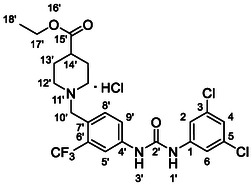


Compound **65** (50 mg, 0.26 mmol, 1.0 eq) was suspended in SOCl_2_ (0.6 mL) and heated to 70 °C for 1 h. The excess of SOCl_2_ was removed under reduced pressure, the residue was dissolved in DCM (5.0 mL), and the solution was added dropwise to a solution of ethyl piperidin‐4‐carboxylate (103 mg, 0.65 mmol, 2.5 eq) in DCM (5.0 mL). After stirring for 30 min, the solvent was removed under reduced pressure, the residue was dissolved in DMF, applied on flash chromatography column, and separated (RP ‐ C18aq, eluent water/acetonitrile, gradient 0‐100%) to obtain the crude intermediate, which was treated with compound **12** (153 mg of the crude mixture, 0.26 mmol, 1.0 eq) according to Method B, followed by Method E, to give **67** (40 mg, 28%) as a white solid. ^1^H NMR (DMSO, 401 MHz) *δ* 10.12 (1H, s, H‐11’), 9.99 (1H, s, H‐3^′^), 9.92 (1H, s, H‐1^′^), 8.10—8.03 (2H, m, H‐5^′^, H‐8^′^), 7.73 (1H, dd, *J* = 8.6, 2.4 Hz, H‐9^′^), 7.54 (2H, d, *J* = 2.0 Hz, H‐2, H‐6), 7.19 (1H, t, *J* = 1.9 Hz, H‐4), 4.37—4.34 (2H, m, H‐10’), 4.08 (2H, q, *J* = 7.1 Hz, H‐17’), 3.42—3.34 (3H, m, H‐12’a), 3.15—3.02 (2H, m, H‐12’b), 2.71—2.60 (1H, m, H‐14’), 2.14—1.87 (4H, m, H‐13’), 1.18 (3H, t, *J* = 7.1 Hz, H‐18’). ^13^C NMR (DMSO, 101 MHz) *δ* 172.96 (C‐15’), 152.30 (C‐2′), 141.88 (C‐1), 140.87 (C‐4^′^), 134.17 (C‐3, C‐5), 134.15 (C‐8^′^), 129.15 (q, *J* = 28.1 Hz, C‐6^′^), 123.71 (q, *J* = 274.7 Hz, CF_3_), 121.62 (C‐9^′^), 121.28 (C‐4), 120.63 (C‐7^′^), 116.30 (C‐2, C‐6), 115.17 (q, *J* = 6.5 Hz, C‐5^′^), 60.32 (C‐17’), 55.37 (C‐10’), 51.30 (C‐18, C‐12’), 37.76 (C‐14’), 25.04 (C‐17, C‐13’), 14.04 (C‐18’). HRMS (ESI+): *m/z* [M + H]^+^ calculated for C_23_H_25_O_3_N_3_Cl_2_F_3_ = 518.1220, found: 518.1216.

##### 1‐(3,5‐Dichlorophenyl)‐3‐(4‐((1,1‐Dioxidothiomorpholino)Methyl)‐3‐(Trifluoromethyl)Phenyl)Urea Hydrochloride (68)


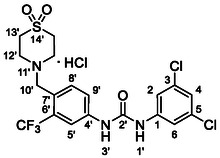


Compound **65** (60 mg, 0.31 mmol, 1.0 eq) was suspended in SOCl_2_ (0.6 mL) and heated to 70 °C for 1 h. The excess of SOCl_2_ was removed under reduced pressure, the residue was dissolved in DCM (5.0 mL), and this solution was added dropwise to a solution of thiomorpholine 1,1‐dioxide (127 mg, 1.26 mmol, 4.0 eq) in DCM (5.0 mL). The mixture was stirred for 30 min, the solvent was removed under reduced pressure, and the residue was dissolved in DMF, applied on flash chromatography column, and separated (RP ‐ C18aq, eluent water/acetonitrile, gradient 0‐100%) to give the crude intermediate, which was treated with compound **12** (222 mg of the crude mixture, 0.38 mmol, 1.2 eq) according to Method B to give **68** (14 mg, 9%) as a yellow solid. ^1^H NMR (DMSO, 500 MHz) *δ* 9.92 (2H, s, H‐1^′^, H‐3^′^), 8.66 (1H, s, H‐11’), 8.00 (1H, d, *J* = 2.3 Hz, H‐5^′^), 7.97—7.89 (1H, m, H‐8^′^), 7.65 (1H, dd, *J* = 8.3, 2.3 Hz, H‐9^′^), 7.53 (2H, d, *J* = 1.9 Hz, H‐2, H‐6), 7.18 (1H, t, *J* = 1.8 Hz, H‐4), 4.37—4.06 (2H, m, H‐10’), 3.52—3.21 (4H, m, H‐13’), 2.94—2.84 (4H, m, H‐12’). ^13^C NMR (DMSO, 126 MHz) *δ* 152.35 (C‐2′), 142.00 (C‐1), 134.16 (C‐3, C‐5), 123.94 (q, *J* = 274.7 Hz, CF_3_), 121.60 (C‐9^′^), 121.14 (C‐4), 116.20 (C‐2, C‐6), 115.07 (q, *J* = 6.3 Hz, C‐5^′^), 55.10 (C‐10’), 50.38 (C‐13’), 41.26 (C‐12’). HRMS (ESI+): *m/z* [M + H]^+^ calculated for C_19_H_19_O_3_N_3_Cl_2_F_3_ = 496.0471, found: 496.0468.

##### Methyl 4‐(4‐(3‐(3,5‐Dichlorophenyl)Ureido)‐2‐(Trifluoromethyl)Benzyl)Morpholine‐2‐Carboxylate Hydrochloride (69)


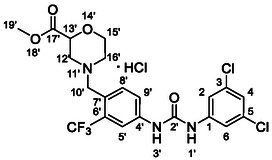


Compound **65** (40 mg, 0.21 mmol, 1.0 eq) was suspended in SOCl_2_ (0.6 mL) and heated to 70 °C for 1 h. The excess of SOCl_2_ was removed under reduced pressure, the residue was dissolved in DCM (5.0 mL), and the solution was added dropwise to a solution of methyl morpholine‐2‐carboxylate hydrochloride (114 mg, 0.63 mmol, 3.0 eq) and DBU (96 mg, 0.63 mmol, 3.0 eq) in DCM (5.0 mL). After 30 min of stirring, the solvent was removed under reduced pressure, and the residue was dissolved in DMF, applied on flash chromatography column, and separated (RP ‐ C18aq, eluent water/acetonitrile, gradient 0–100%) to obtain the crude intermediate, which was treated with compound **12** (148 mg of the crude mixture, 0.25 mmol, 1.0 eq) according to Method B, followed by Method E, to give **69** (25 mg, 22%) as a yellowish oil. ^1^H NMR (DMSO, 401 MHz) *δ* 9.24 (1H, s, H‐3^′^), 9.18 (1H, s, H‐1^′^), 7.95 (1H, t, *J* = 1.3 Hz, H‐5^′^), 7.63—7.60 (2H, m, H‐8^′^, H‐9^′^), 7.55 (2H, d, *J* = 1.9 Hz, H‐2, H‐6), 7.18 (1H, t, *J* = 1.9 Hz, H‐4), 4.24 (1H, dd, *J* = 7.1, 3.1 Hz, H‐13’), 3.89 (1H, ddd, *J* = 11.3, 5.4, 3.1 Hz, H‐15’a), 3.64 (3H, s, H‐19’), 3.61—3.53 (3H, m, H‐10’, H‐15’b), 2.70—2.62 (1H, m, H‐12’a), 2.47—2.30 (3H, m, H‐12’b, H‐16’). ^13^C NMR (DMSO, 101 MHz) *δ* 170.14 (C‐17’), 152.28 (C‐2′), 142.02 (C‐1), 138.59 (C‐4^′^), 134.10 (C‐3, C‐5), 131.57 (C‐8^′^), 129.68 (C‐7^′^), 127.74 (q, *J* = 29.7 Hz, C‐6^′^), 124.27 (q, *J* = 274.0 Hz, CF_3_), 121.94 (C‐9^′^), 121.18 (C‐4), 116.56 (C‐2, C‐6), 115.56 (q, *J* = 6.3 Hz, C‐5^′^), 73.19 (C‐13’), 64.61 (C‐15’), 57.43 (C‐10’), 54.14 (C‐12’), 52.44 (C‐16’), 51.64 (C‐19’). HRMS (ESI+): *m/z* [M + H]^+^ calculated for C_21_H_21_O_4_N_3_Cl_2_F_3_ = 506.0856, found: 506.0851.

##### 1‐(3,5‐Dichlorophenyl)‐3‐(4‐((3‐Oxopiperazin‐1‐Yl)Methyl)‐3‐(Trifluoromethyl)Phenyl)Urea Hydrochloride (70)


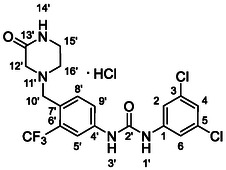


Compound **65** (50 mg, 0.26 mmol, 1.0 eq) was suspended in SOCl_2_ (1.0 mL) and heated to 70 °C for 1 h. The excess of SOCl_2_ was removed under reduced pressure, the residue was dissolved in DCM (5.0 mL), and the solution was added dropwise to a solution of 2‐oxopiperazine (130 mg, 1.31 mmol, 5.0 eq) in DCM (5.0 mL). After 30 min of stirring, the solvent was removed under reduced pressure, and the residue was dissolved in DMF, applied on flash chromatography column, and separated (RP ‐ C18aq, eluent water/acetonitrile, gradient 0‐100%) to obtain the crude intermediate, which was treated with compound **12** (184 mg of crude mixture, 0.31 mmol, 1.2 eq) according to Method B, followed by Method E, to give **70** (74 mg, 61%) as a white solid. ^1^H NMR (DMSO, 401 MHz) *δ* 9.98 (1H, s, H‐3^′^), 9.91 (1H, s, H‐1^′^), 8.36 (1H, s, H‐14’), 8.06 (1H, d, *J* = 2.3 Hz, H‐5^′^), 8.01 (1H, d, *J* = 8.5 Hz, H‐8^′^), 7.72 (1H, dd, *J* = 8.6, 2.3 Hz, H‐9^′^), 7.53 (2H, d, *J* = 1.9 Hz, H‐2, H‐6), 7.18 (1H, t, *J* = 1.9 Hz, H‐4), 4.46—4.37 (2H, m, H‐12’), 3.81—3.67 (2H, m, H‐10’), 3.45—3.36 (2H, m, H‐16’), 3.34—3.25 (2H, m, H‐15’). ^13^C NMR (DMSO, 101 MHz) *δ* 152.32 (C‐2′), 141.91 (C‐1), 140.80 (C‐4^′^), 134.19 (C‐3, C‐5), 133.90 (C‐8^′^), 129.08 (q, *J* = 30.0 Hz, C‐6^′^), 123.73 (q, *J* = 274.3 Hz, CF_3_), 121.66 (C‐9^′^), 121.28 (C‐4), 116.31 (C‐2, C‐6), 115.20 (q, *J* = 6.3 Hz, C‐5^′^), 54.85 (C‐12’), 53.01 (C‐10’), 46.96 (C‐15’), 37.31 (C‐16’). HRMS (ESI‐): *m/z* [M—H]^−^ calculated for C_19_H_18_O_2_N_4_Cl_2_F_3_ = 461.0753, found: 461.0751.

##### Lithium 4‐(4‐(3‐(3,5‐Dichlorophenyl)Ureido)‐2‐(Trifluoromethyl)Benzyl)Morpholine‐2‐Carboxylate (71)


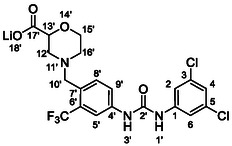


Compound **69** (200 mg, 0.40 mmol, 1.0 eq) was dissolved in a mixture of water (2.0 mL) and dioxane (2.0 mL), and lithium hydroxide monohydrate (50 mg, 1.20 mmol, 3.0 eq) was added at RT. The mixture was stirred at RT for 30 min, applied on a flash chromatography column, and separated (RP ‐ C18aq, eluent water/acetonitrile, gradient 0‐100%) to obtain **71** (190 mg, 98%) as a yellowish solid. ^1^H NMR (DMSO, 401 MHz) *δ* 12.25 (1H, s, H‐18’), 12.19 (1H, s), 8.26 (1H, d, *J* = 2.2 Hz, H‐5^′^), 7.68 (2H, d, *J* = 1.9 Hz, H‐2, H‐6), 7.54 (1H, d, *J* = 8.4 Hz, H‐8^′^), 7.37—7.31 (1H, m, H‐9^′^), 7.07 (1H, t, *J* = 1.9 Hz, H‐4), 4.02 (1H, d, *J* = 10.6 Hz, H‐15’a), 3.81—3.68 (2H, m, H‐10’a, H‐13’), 3.58—3.48 (1H, m, H‐15’b), 3.28 (1H, d, *J* = 13.9 Hz, 10’b), 2.74—2.65 (1H, m, H‐12’b), 2.58—2.52 (1H, m, H‐16’a), 2.37—2.20 (2H, m, H‐12’b, H‐16’b). ^13^C NMR (DMSO, 101 MHz) *δ* 175.32 (C‐17’), 153.36 (C‐2′), 143.68 (C‐1), 139.80 (C‐4^′^), 133.90 (C‐3, C‐5), 131.60 (q, *J* = 3.4 Hz, C‐8^′^), 128.89 (C‐7^′^), 127.67 (q, *J* = 29.5 Hz, C‐6^′^), 124.54 (q, *J* = 274.2 Hz, CF_3_), 121.54 (C‐9^′^), 120.00 (C‐4), 115.91 (C‐2, C‐6), 115.47 (C‐5^′^), 75.99 (C‐13’), 64.39, 57.94 (C‐10’), 55.25 (C‐12’), 53.72 (C‐16’). HRMS (ESI+): *m/z* [M + H]^+^ calculated for C_20_H_19_O_4_N_3_Cl_2_F_3_ = 492.0699, found: 492.0697.

##### 4‐(4‐(3‐(3,5‐Dichlorophenyl)Ureido)‐2‐(Trifluoromethyl)Benzyl)Morpholine‐2‐Carboxamide Hydrochloride (72)


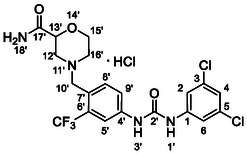


Compound **71** (50 mg, 0.10 mmol, 1.0 eq), ammonium chloride (27 mg, 0.50 mmol, 5.0 eq), DBU (0.05 mL, 0.30 mmol, 3.0 eq), and HATU (50 mg, 0.13 mmol, 1.3 eq) were treated according to Method C, followed by Method E, to give **72** (36 mg, 72%) as a yellowish solid. ^1^H NMR (DMSO, 401 MHz) *δ* 9.24 (2H, s, H‐18’), 7.96 (1H, d, *J* = 2.1 Hz, H‐5^′^), 7.69—7.59 (2H, m, H‐8^′^, H‐9^′^), 7.55 (2H, d, *J* = 1.9 Hz, H‐2, H‐6), 7.18 (1H, t, *J* = 1.9 Hz, H‐4), 3.95—3.77 (2H, m, H‐13’, H‐15’a), 3.65—3.49 (3H, m, H‐10’, H‐15’b), 2.93—2.83 (1H, m, H‐12’a), 2.67—2.60 (1H, m, H‐16’a), 2.22—2.11 (1H, m, H‐16’b), 2.00—1.86 (1H, m, H‐12’b). ^13^C NMR (DMSO, 101 MHz) *δ* 171.38 (C‐17’), 152.31 (C‐2′), 142.09 (C‐4^′^), 138.60 (C‐1), 134.09 (C‐3, C‐5), 131.67 (C‐8^′^), 129.83 (C‐7^′^), 127.72 (q, *J* = 29.3 Hz, C‐6^′^), 124.30 (q, *J* = 274.3 Hz, CF_3_), 122.03 (C‐9^′^), 121.14 (C‐4), 116.57 (C‐2, C‐6), 115.50 (q, *J* = 6.5 Hz, C‐5^′^), 75.32 (C‐13’), 65.90 (C‐15’), 57.49 (C‐10’), 54.76 (C‐12’), 52.46 (C‐16’). HRMS (ESI+): *m/z* [M + H]^+^ calculated for C_20_H_20_O_3_N_4_Cl_2_F_3_ = 491.0859, found: 491.0856.

##### Biology: Protein Expression

Vectors for expression of 6×His‐TEV‐nsp7 (#154757), 6×His‐TEV‐nsp8 (#154758) and 6×HisMBP‐TEV‐nsp12 (#154759) were ordered from AddGene [Hillen et al. 2020].

6×His‐TEV‐Nsp7 and 6×His‐TEV‐Nsp8 were separately expressed in *E. coli* BL21 CodonPlus RIL. The cell culture was grown at 37 °C until reaching OD_600_ 0.6, after which the expression was induced by the addition of Isopropyl *β*‐D‐1‐thiogalactopyranoside to the final concentration of 0.4 mM. Bacterial cultures were then incubated for another 16 h at 18 °C. Afterward, the cells were harvested by centrifugation at 3,000 × g for 15 min and resuspended in 50 mM Na‐HEPES, pH 7.4, 300 mM NaCl, 30 mM imidazole, 10% (v/v) glycerol and 2 mM *β*‐mercaptoethanol with the addition of benzonase and protease inhibitor cocktail (Roche). The cells were then disrupted using a one‐shot homogenizer (Constant Systems) operating at 1.9 kbar. The obtained lysate was clarified by centrifugation at 50,000 × g for 30 min and loaded onto HisTrap HP column (Cytiva) equilibrated in lysis buffer. The column was subsequently washed with a high‐salt buffer containing 1 M NaCl and a low‐salt buffer with 150 mM NaCl. Finally, the his‐tagged proteins were eluted by 500 mM imidazole. To cleave off the purification tag, his‐tagged TEV protease was added in a 1:10 (w:w) ratio, and the whole mixture was dialyzed against the low‐salt buffer for 16 h at 4 °C. Reverse IMAC was subsequently utilized to purify nsp7 or nsp8 of uncleaved constructs, TEV protease, and cleaved purification tags. The flow through fractions were dialyzed against storage buffer (20 mM Na‐HEPES pH 7.4, 150 mM NaCl, 5% (v/v) glycerol, 1 mM TCEP). The proteins were finally concentrated to 484 µM for nsp7 and 221 µM for nsp8 using an Amicon Ultra Centrifugal Filter, 3 kDa MWCO (Millipore) and stored at −80 °C.

*Sf9* insect cell line was employed to produce nsp12. Bac‐to‐Bac system (Invitrogen) was utilized to generate the 6×His‐MBP‐TEV‐nsp12 bacmid, after which the insect cells were then transfected, and the supernatant containing the recombinant baculoviruses was harvested after 5 days. The expression was initiated by infecting the cell suspension (3 × 10^6^ cells/ml) with the stock solution of baculoviruses in a 1:100 ratio. Subsequently, the culture was incubated at 27 °C for 96 h. After expression, the cells were harvested by centrifugation at 3000 × g for 15 min, resuspended in a lysis buffer containing 50 mM Na‐HEPES pH 7.4, 300 mM NaCl, 30 mM imidazole, 3 mM MgCl_2_ 10% (v/v) glycerol, and 5 mM *β*‐mercaptoethanol, and disrupted by one‐shot homogenizer (Constant Systems). Before disruption, benzonase and protease inhibitor cocktail (Roche) were added. The lysate was subsequently cleared by centrifugation at 70,000 × g for 30 min and ultracentrifugation at 235,000 × g for 60 min and applied onto an HisTrap HP column (Cytiva) in lysis buffer. The column was washed with buffer containing 1 M NaCl. An MBPTrap column (Cytiva) was then connected downstream to the HisTrap column, and both columns were washed with 500 mM imidazole to elute His‐tagged proteins onto the MBPTrap. The HisTrap column was subsequently disconnected, and the MBPTrap was washed with additional 5 column volumes of lysis buffer. Upon elution with 116.9 mM maltose, 6×His tagged TEV protease was added in a 1:10 (w:w) ratio, and the sample was dialyzed against 3 liters of lysis buffer for 16 h at 4 °C. Uncleaved protein, 6×His‐MBP tag, and protease were subsequently removed on an HisTrap column, and the flowthrough fraction containing nsp12 was diluted to 80 mM NaCl using heparin dilution buffer (20 mM Na‐HEPES pH 7.4, 1 mM MgCl_2_, 10% (v/v) glycerol, 5 mM *β*‐mercaptoethanol). The sample was then loaded onto HiTrap Heparin HP (Cytiva) equilibrated in heparin binding buffer containing 80 mM NaCl. Nsp12 was eluted with a linear gradient from 80 mM to 1 M NaCl. Peak fractions were pooled and dialyzed against 3 liters of storage buffer (20 mM Na‐HEPES pH 7.4, 300 mM NaCl, 1 mM MgCl_2_, 10% (v/v) glycerol, 1 mM TCEP). Finally, the sample was concentrated in an Amicon Ultra Centrifugal Filter, 50 kDa MWCO (Millipore) to the final concentration of 21.5 µM and stored at −80 °C as single‐use aliquots.

##### The Fluorescence‐Based RdRp Polymerase Assay

The activity of the RNA‐dependent RNA polymerase complex in real time was monitored using a SYBR Green I‐based approach. First, the reaction mixture was prepared containing reaction buffer (20 mM HEPES pH 8, 10 mM KCl, 6 mM MgCl_2_, 0.01% Triton X‐100, 1 mM DTT), 250 µM ATP, 5 µM SYBR Green I, and RdRp complex composed of nsp12, nsp7, and nsp8 at the ratio of 1:3:3 (nsp12:nsp7:nsp8) to the final concentration of 0.63 µM:1.88 µM:1.88 µM. The reaction mixture was distributed in 17 µl aliquots into a 96‐well plate. For the inhibitor testing, 1 µl of the compound solution or solvent, to ensure the equal 5% concentration of DMSO in both the controls and testing reactions. Reactions were then initiated by adding 2µl of dsRNA solution to the final concentration of 0.5 µM. The dsRNA solution was prepared by annealing of ssRNA template (5′‐ _58_UAUAACUUAAUCUCACAUAGC—3′) and ssRNA primer (5′‐ GCUAUGUGAGAUUAAGUUAU—3′). All components of the annealing reaction mixture, namely annealing buffer (10 mM Na‐HEPES pH 8.0, 25 mM NaCl, 2.5 mM EDTA), ssRNA template, and ssRNA primer, were mixed and heated for 5 min at 94 °C. The mixture was then slowly cooled to room temperature and stored at −80 °C. Reactions were monitored using a thermocycler QuantStudio 5 real‐time PCR system (Applied Biosystems) for 50 min at 30 °C. The fluorescence increase resulting from SYBR Green I binding to the nascent dsRNA molecule was measured every minute and plotted over time.

Each measurement was performed in two technical replicates. After background subtraction, the area under the curve (AUC) for each reaction was determined using the midpoint method for numerical integration. The residual RdRp activity (RA) in % was obtained as AUC_i_/AUC_*n*_ × 100, where AUC_i_ and AUC_*n*_ represent AUC for reactions with and without an inhibitor, respectively. Standard deviation of each RA was calculated by propagating the error of the ratio, thereby considering both the error in AUC_i_ and AUC_*n*_. The RdRp polymerase activity was verified previously in a gel‐based RNA polymerase assay.^[10]^

##### Antiviral and Cytotoxicity Assays

The anti‐SARS‐CoV‐2 activity was measured by determining the extent to which the test compounds inhibited virus‐induced cytopathic effect in Calu‐3 cells. Briefly, twofold serial dilutions of compounds were pipetted in triplicates into a 384‐well plate containing 15,000 Calu‐3 cells/well in DMEM medium with 2% FBS, 100 U of penicillin/ml, and 100 μg of streptomycin/ml (all Merck). After 1 h of incubation, SARS‐CoV‐2 was added at multiplicity of infection 0.01. Following 3 days of incubation at 37 °C in 5% CO_2_ atmosphere, the cell viability was determined by colorimetric assay using XTT (2,3‐bis‐(2‐methoxy‐4‐nitro‐5‐sulfophenyl)‐2H‐tetrazolium‐5‐carboxanilide, Sigma–Aldrich) for 4 h, and the absorbance was measured using EnVision plate reader (Perkin Elmer). Drug concentrations required to reduce viral cytopathic effect by 50% (EC_50_) were calculated using nonlinear regression from plots of percentage cell viability versus log_10_ drug concentration using GraphPad Prism v.9.0.0 (GraphPad Software).

Cytotoxicity was evaluated by incubating the same two‐fold serial dilutions of each compound with Calu‐3 cells as above but without the virus. Following 3 days of incubation at 37 °C in 5% CO_2_, the cell viability was determined by the addition of XTT solution as above. The compound cytotoxic concentrations resulting in a 50% reduction of absorbance (CC_50_) were calculated from plots of the percentage of absorbance versus log10 of drug concentration as above.

##### Molecular Docking and Molecular Dynamics Simulations

Trial molecular dynamics simulations were performed using Schrödinger Drug Discovery Suite version 2018–4 (Schrödinger, LLC, New York, NY, 2018). The structure of SARS‐CoV‐2 RdRp (PDB ID: 7ED5) was prepared for docking by Protein Preparation Wizard. It was protonated (neutral pH) and its energy was minimized.

The docking study was performed using the Molecular Operating Environment software (MOE).^[^ ^16^ ^]^ Crystal structures of the receptors 7ED5 was prepared with MOE QuickPrep tool with default setup; the structure was not minimized. Structures of all final compounds were properly protonated and minimized to RMS gradient of 0.001. For docking studies, rigid dock protocol was used, with structure waters excluded and ligand bond rotation enabled. The default placement and refinement method was used with 50 retained structures after the first refinement and 10 retained structures after the second refinement. For all calculations, Amber 10:EHT mixed force field was used with R‐Field solvent model. A long simulation was performed in Gromacs. Simulation details, inputs, and trajectories are available via Zenodo (DOI 10.5281/zenodo.15461444).

## Conflict of Interest

The authors declare no conflict of interest.

## Data Availability

The data that support the findings of this study are available from the corresponding author upon reasonable request.

## References

[1] A. Haileamlak , Ethiop. J. Health Sci. 2021, 31, 1073. 35392335 10.4314/ejhs.v31i6.1 PMC8968362

[2] A. Bhimraj , R. L. Morgan , A. H. Shumaker , L. R. Baden , V. C.‐C. Cheng , K. M. Edwards , J. C. Gallagher , R. T. Gandhi , W. J. Muller , M. M. Nakamura , J. C. O’Horo , R. W. Shafer , S. Shoham , M. H. Murad , R. A. Mustafa , S. Sultan , Y. Falck‐Ytter , Clin. Infect. Dis. 2024, 78, e250. 36063397 10.1093/cid/ciac724 PMC9494372

[3] T. K. Burki , Lancet Respir. Med. 2022, 10, e18.35033223

[4] Y.‐S. Wu , W.‐H. Lin , J. T.‐A.Hsu , H.‐P. Hsieh , Curr. Med. Chem. 2006, 13, 2003. 16842194 10.2174/092986706777584988

[5] Y. Wang , V. Anirudhan , R. Du , Q. Cui , L. Rong , J. Med. Virol. 2021, 93, 300. 32633831 10.1002/jmv.26264

[6] Y. Ratan , A. Rajput , V. Jain , D. K. Mishra , R. K. Gautam , A. Pareek , Curr. Pharm. Biotechnol. 2023, 24, 1727. 36861800 10.2174/1389201024666230302113110

[7] R. Alipoor , R. Ranjbar , Biol. Chem. 2023, 404, 569. 36490203 10.1515/hsz-2022-0323

[8] L. Zhao , S. Li , W. Zhong , Front. Pharmacol. 2022, 13, 840639. 35281901 10.3389/fphar.2022.840639 PMC8916227

[9] E. C. Smith , H. Blanc , M. C. Surdel , M. Vignuzzi , M. R. Denison , PLoS Pathog. 2013, 9, e1003565. 23966862 10.1371/journal.ppat.1003565 PMC3744431

[10] A. Chayka , M. Danda , A. Dostálková , V. Spiwok , A. Klimešová , M. Kapisheva , M. Zgarbová , J. Weber , T. Ruml , M. Rumlová , Z. Janeba , ChemMedChem 2024, 19, e202400367. 39140451 10.1002/cmdc.202400367 PMC11617668

[11] B. Das , A. T. K. Baidya , A. T. Mathew , A. K. Yadav , R. Kumar , Bioorg. Med. Chem. 2022, 56, 116614. 35033884 10.1016/j.bmc.2022.116614

[12] A. R. Renslo , H. Gao , P. Jaishankar , R. Venkatachalam , M. F. Gordeev , Org. Lett. 2005, 7, 2627. 15957907 10.1021/ol050730h

[13] A. Shannon , V. Fattorini , B. Sama , B. Selisko , M. Feracci , C. Falcou , P. Gauffre , P. E. Kazzi , A. Delpal , E. Decroly , K. Alvarez , C. Eydoux , J.‐C. Guillemot , A. Moussa , S. S. Good , P. La Colla , K. Lin , J.‐P. Sommadossi , Y. Zhu , X. Yan , H. Shi , F. Ferron , B. Canard , Nat. Commun. 2022, 13, 621. 35110538 10.1038/s41467-022-28113-1 PMC8810794

[14] G. M. Allan , N. Vicker , H. R. Lawrence , H. J. Tutill , J. M. Day , M. Huchet , E. Ferrandis , M. J. Reed , A. Purohit , B. V. L. Potter , Bioorg. Med. Chem. 2008, 16, 4438. 18329273 10.1016/j.bmc.2008.02.059

[15] M. A. Walker , Tetrahedron 1997, 53, 14591.

[16] Molecular Operating Environment (MOE) , 2024 Chemical Computing Group ULC, 910-1010 Sherbrooke St. W., Montreal, QC H3A 2R7 2025.

